# The epigenetic etiology of cardiovascular disease in a longitudinal Swedish twin study

**DOI:** 10.1186/s13148-021-01113-6

**Published:** 2021-06-24

**Authors:** Xueying Qin, Ida K. Karlsson, Yunzhang Wang, Xia Li, Nancy Pedersen, Chandra A. Reynolds, Sara Hägg

**Affiliations:** 1 Department of Medical Epidemiology and Biostatistics, Karolinska Institutet, Nobels väg 12A, 17177 Stockholm, Sweden; 2 Department of Epidemiology and Biostatistics, School of Public Health, Peking University, 38# Xueyuan Road, Beijing, 100191 China; 3 Institute of Gerontology and Aging Research Network – Jönköping (ARN-J), School of Health and Welfare, Jönköping University, Jönköping, Sweden; 4 Department of Psychology, University of Southern California, Los Angeles, CA USA; 5 Department of Psychology, University of California, Riverside, USA

**Keywords:** Cardiovascular disease, DNA methylation, Cross-lagged effect, Mediation

## Abstract

**Background:**

Studies on DNA methylation have the potential to discover mechanisms of cardiovascular disease (CVD) risk. However, the role of DNA methylation in CVD etiology remains unclear.

**Results:**

We performed an epigenome-wide association study (EWAS) on CVD in a longitudinal sample of Swedish twins (535 individuals). We selected CpGs reaching the Bonferroni-corrected significance level (2 $\times$ 10^–7^) or the top-ranked 20 CpGs with the lowest *P* values if they did not reach this significance level in EWAS analysis associated with non-stroke CVD, overall stroke, and ischemic stroke, respectively. We further applied a bivariate autoregressive latent trajectory model with structured residuals (ALT-SR) to evaluate the cross-lagged effect between DNA methylation of these CpGs and cardiometabolic traits (blood lipids, blood pressure, and body mass index). Furthermore, mediation analysis was performed to evaluate whether the cross-lagged effects had causal impacts on CVD. In the EWAS models, none of the CpGs we selected reached the Bonferroni-corrected significance level. The ALT-SR model showed that DNA methylation levels were more likely to predict the subsequent level of cardiometabolic traits rather than the other way around (numbers of significant cross-lagged paths of methylation → trait/trait → methylation were 84/4, 45/6, 66/1 for the identified three CpG sets, respectively). Finally, we demonstrated significant indirect effects from DNA methylation on CVD mediated by cardiometabolic traits.

**Conclusions:**

We present evidence for a directional association from DNA methylation on cardiometabolic traits and CVD, rather than the opposite, highlighting the role of epigenetics in CVD development.

**Supplementary Information:**

The online version contains supplementary material available at 10.1186/s13148-021-01113-6.

## Background

Cardiovascular disease (CVD) refers to a group of disorders that affect the heart or blood vessels, of which the most common types are coronary heart disease and stroke [1]. CVD is a global public health concern, many risk factors have been associated with CVD, and new factors emerge with the development of omics technologies, such as genetic variation and epigenetic modifications.

Epigenetics is defined as chemical modifications to the DNA, which regulate gene expression without changing the DNA sequence itself. DNA methylation is a type of epigenetic process where a methyl group is added to the 5′-position of a cytosine, forming 5-methylcytosine. It is mostly found in regions containing a large number of cytosine 5′ to guanine dinucleotides (CpGs) in promoters. As a consequence, gene transcription could be turned off if the CpG is methylated. Due to the advances of new technologies to detect DNA methylation in the last decades [2], especially array-based approaches, it is feasible to assess hundreds of thousands of CpGs along the genome in the population.

Epigenetic studies on DNA methylation further help us to understand how genetic and environmental factors interact at the cellular level to contribute to CVD development. Studies have revealed that DNA methylation is associated with CVD [3–5] and its cardiometabolic risk factors such as body mass index (BMI) [6], lipids [7, 8], and blood pressure (BP) [9]. Furthermore, DNA methylation processes may be involved in the biological mechanisms underlying CVD, such as atherosclerosis [10] and inflammation [11]. However, the underlying mechanisms of these associations are not yet well understood, for example, how DNA methylation co-varies with complex cardiometabolic traits in a longitudinal perspective, and how this co-varying pattern could determine CVD.

Hence, the aim of our study was to perform an epigenome-wide association study (EWAS) of CVD based on a longitudinal sample of Swedish twins. In particular, we aim to (1) explore the association between CpGs and CVD, (2) investigate the association between DNA methylation of CVD-related CpGs and other cardiometabolic traits, especially the co-varying patterns, and (3) examine the mediation path from DNA methylation to cardiometabolic traits and CVD.

## Results

### Characteristics of the study population

This study was based on the Swedish Adoption/Twin Study of Aging (SATSA) [12], a twin-based longitudinal study collecting information on aging-related phenotypes in repeated waves of assessments between 1984 and 2014 in Sweden. Assessment in each wave included a questionnaire survey and an in-person testing (IPT), and each IPT is referred to by adding a number suffix to the assessment name, for example, IPT3 means the third IPT (“Methods” section, Study population). The EWAS was conducted in 535 individuals, including 83 monozygotic (MZ) twin pairs, 155 dizygotic (DZ) twin pairs, and 59 single twins. For the 535 individuals, 187 individuals were classified as MZ, 347 were classified as DZ, and one has unknown zygosity. There were totally 1399 DNA methylation measurements, and half of the participants (269/535) had at least three measurements of DNA methylation. Two hundred and twelve individuals were diagnosed with non-stroke CVD in the registry database, 108 were diagnosed with stroke regardless of subtype, and 85 of them were specifically diagnosed with ischemic stroke. The general characteristics are displayed in Table 1. Information on DNA methylation samples and cardiometabolic traits (lipids, BMI, BP) in different IPTs is presented in Additional file 1: Table S1.

**Table 1:** Characteristics of the study sample

Characteristics	*N* or mean value
Individuals (MZ pairs, DZ pairs, single twins)	535 (83, 155, 59)
*Zygosity*	
MZ	187
DZ	347
Unknown	1
*Repeated measures of DNA methylation per person*	
1	145
2	121
3	119
4	98
5	49
6	3
Female (%)	313 (58.5)
Baseline age (years), mean (SD)	72.7 (9.3)
Baseline current smokers (%)	85 (15.9)
Non-stroke CVD (%)	212 (39.6)
Overall stroke (%)	108 (20.2)
Ischemic stroke (%)	85 (15.9)
Statin user (%)	95 (18.0)

*MZ* monozygotic twins, *DZ* dizygotic twins, *CVD* cardiovascular disease, *SD* standard deviation

### Identification of epigenome-wide CpGs associated with CVD

All the analysis was performed in all sample, MZ and DZ samples, respectively. It meant the result in all sample if there was no particular noting. In EWAS analysis of all sample, for each category of CVD events, denoted as non-stroke CVD, overall stroke, and ischemic stroke, no CpGs reached Bonferroni-corrected significance (*P* < 2 $\times$ 10^–7^); therefore, we identified 20 CpGs with the lowest *P* value in a model adjusted for age, sex, and smoking in relation to the three CVD events, respectively. Seven CpGs were associated with both overall stroke and ischemic stroke (Additional file 1: Table S2). Manhattan plots show associations distributed across the whole genome (Additional file 2: Figure S1, Additional file 3: Figure S2, Additional file 4: Figure S3). In the separate analysis for MZ and DZ, the association between cg11188837 and ischemic stroke was significant in DZ (effect size = −0.0942, *P* value = 8.70 $\times$ 10^–9^) (Additional file 1: Table S2).

We did not find significant associations (*P* value was set to 6.25 $\times$ 10^–4^ for the multiple testing) between age at the onset of CVD and DNA methylation of these CVD-related CpGs (Additional file 1: Tables S3–S8) in all sample, MZ and DZ samples.

### Association between DNA methylation of CVD-related CpGs and cardiometabolic traits

**Fig. 1:**
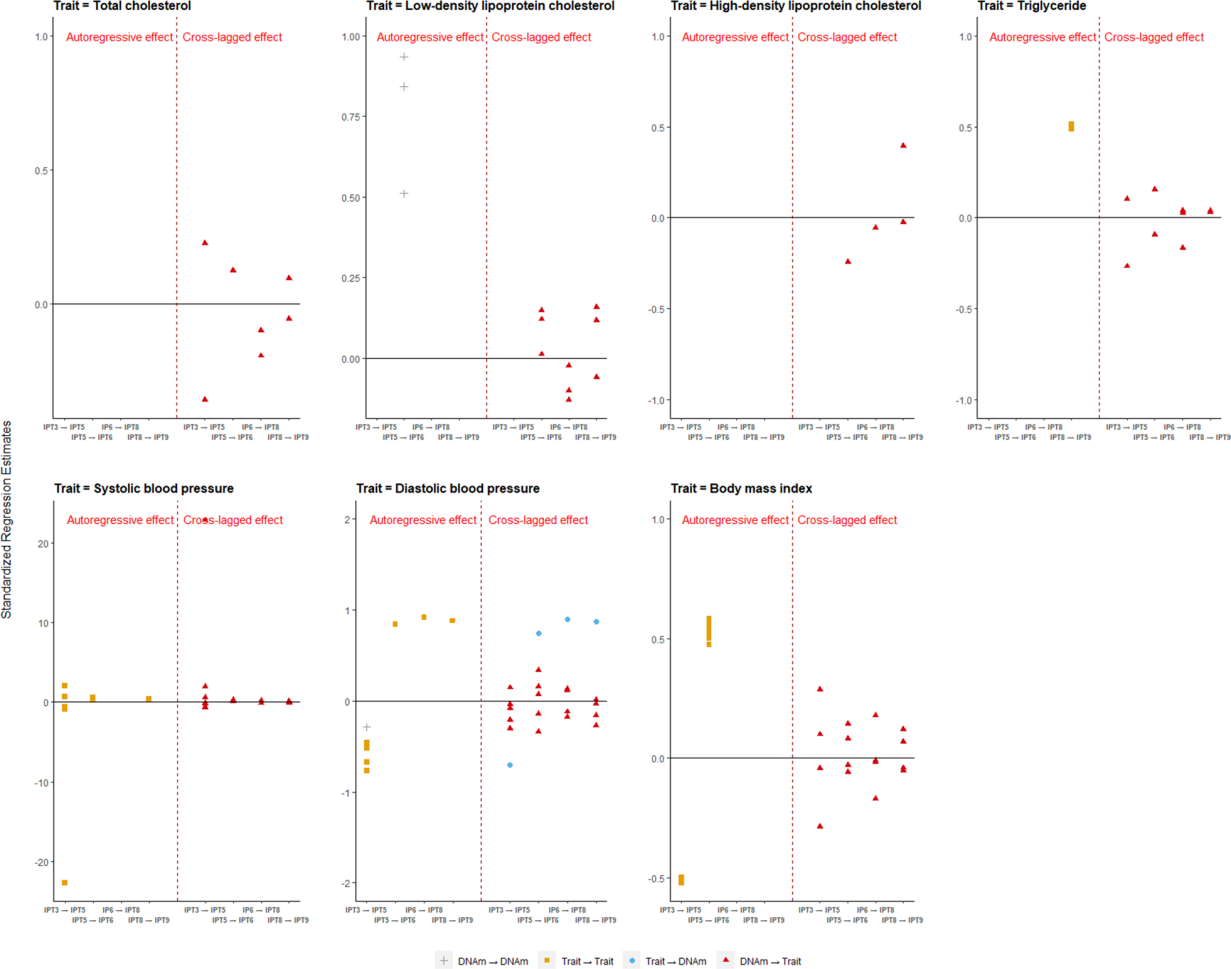
Autoregressive and cross-lagged effect between DNA methylation of non-stroke CVD-related CpGs and cardiometabolic traits in all sample. Each point represents one significant effect (*P* value was set to 3 $\times$ 10^–4^). The *X*-axis represents the effect at different adjacent time points, for example, IPT3 → IPT5 means the effect of one variable at IPT3 on the other variable at IPT5. The *Y*-axis represents the standardized estimation coefficient from the regression model. The left part of the figure is the autoregressive effect, and the right part is the cross-lagged effect. “DNAm → DNAm” (gray plus sign) represents autoregressive effect of DNA methylation, “Trait → Trait” (blue circle) represents autoregressive effect of trait, “Trait → DNAm”(brown square) represents cross-lagged effect from trait to DNA methylation, and “DNAm → Trait” (red triangle) represents cross-lagged effect from DNA methylation to trait

Next, we applied a bivariate autoregressive latent trajectory model with structured residuals (ALT-SR; “Methods” section, Statistical analysis) [13] to examine the autoregressive and cross-lagged effect for identified CpGs on seven cardiometabolic traits (total cholesterol (TC), low-density lipoprotein cholesterol (LDL), high-density lipoprotein cholesterol (HDL), total triglyceride (TG), systolic blood pressure (SBP), diastolic blood pressure (DBP), and BMI). The autoregressive effect describes the within-person changes of each of the two constructs over time, and the cross-lagged effect describes whether DNA methylation at one time point predicts within-person changes of cardiometabolic traits at an adjacent later time point, and/or vice versa. In ALT-SR for non-stroke CVD-related CpGs (*P* value was set to 0.05/(20 × 7) $\approx$ 3 $\times$ 10^–4^ after Bonferroni adjustment based on the number of CpGs and traits), we observed 162 significant autoregressive and cross-lagged associations, of which 84 associations were cross-lagged effects from DNA methylation measurements prior to cardiometabolic trait measurements and four were opposite cross-lagged effects. More autoregressive associations were found for cardiometabolic traits than DNA methylation (70 vs. 4), and most of them in TG, BMI, SBP, and DBP. In particular, we found that some CpGs had cross-lagged effects on multiple traits at multiple time points, for example, cg01417615 had cross-lagged effects for all lipids and BMI, cg02268354 had effects on TC, LDL, TG, BMI, and DBP, cg04482923 had effects on LDL, HDL, and BMI, cg20729301 had effects on BMI, SBP, and DBP, and cg24610274, cg11521799 and cg01207734 had cross-lagged effects on both SBP and DBP. The four cross-lagged effects detected in opposite direction, trait on DNA methylation, were found for DBP at IPT3 through IPT8 determining DNA methylation of cg05367173 at the subsequent time points. This site was found to have bidirectional associations with DBP since it also had cross-lagged effects on DBP at IPT3 and IPT5. Most of the models fit the data well, but there were some exceptions for models of TG, SBP, and DBP. In separate analysis for MZ and DZ, ALT-SR model failed to converge in a few combinations of CpGs and cardiometabolic traits due to the small sample size and model complexity; therefore, there was no output for these conditions. For those converged models in both all sample and subsamples, there were no big differences for the cross-lag effect pattern between CpGs and cardiometabolic traits in different samples. (Fig. 1, Additional file 5: Figure S4, Additional file 6: Figure S5, Additional file 1: Table S9A–S9G).

**Fig. 2:**
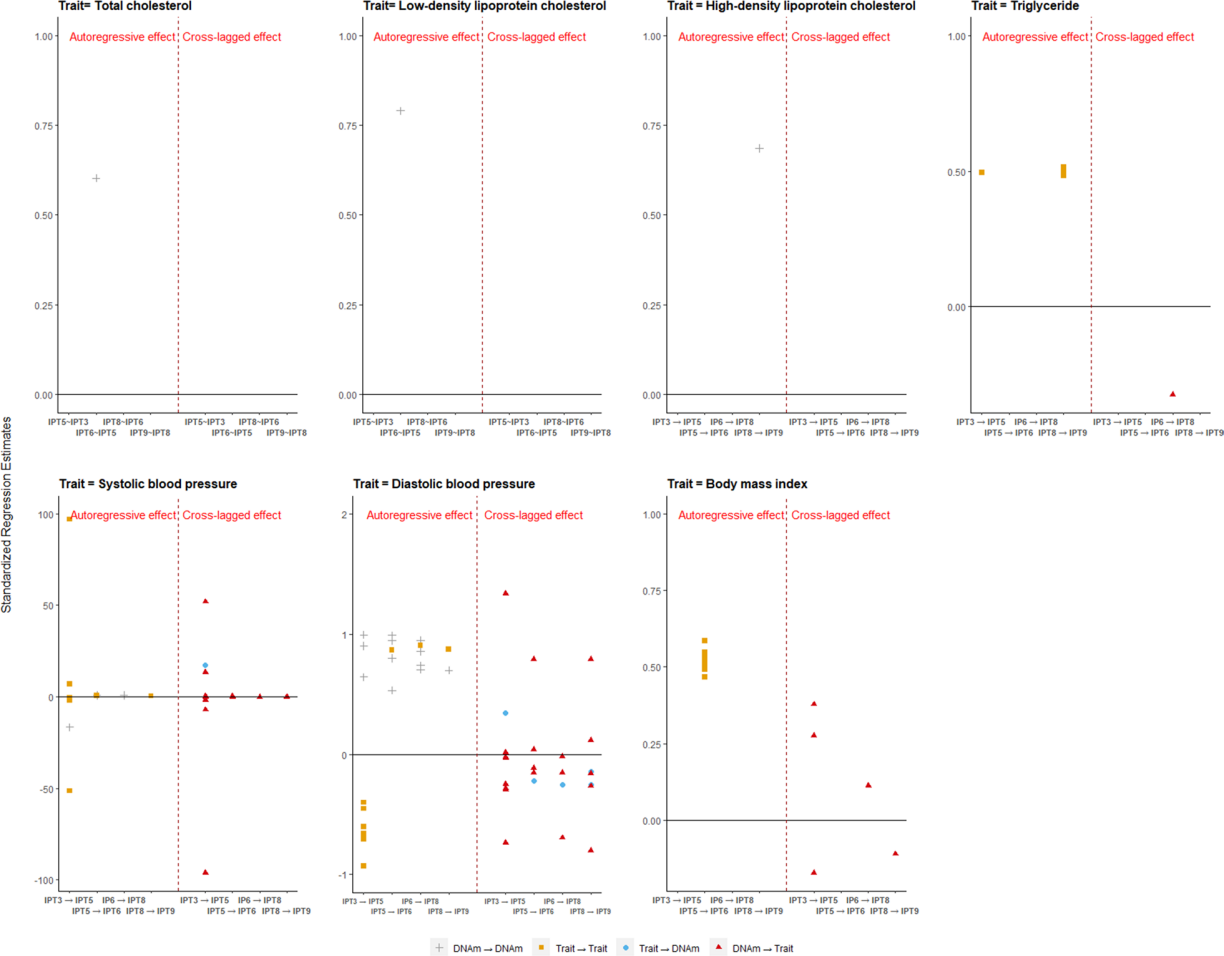
Autoregressive and cross-lagged effect between DNA methylation of overall stroke-related CpGs and cardiometabolic traits in all sample. Each point represents one significant effect (*P* value was set to 3 $\times$ 10^–4^). The *X*-axis represents the effect at different adjacent time points, for example, IPT3 → IPT5 means the effect of one variable at IPT3 on the other variable at IPT5. The *Y*-axis represents the standardized estimation coefficient from the regression model. The left part of the figure is the autoregressive effect, and the right part is the cross-lagged effect. “DNAm → DNAm” (gray plus sign) represents autoregressive effect of DNA methylation, “Trait → Trait” (blue circle) represents autoregressive effect of trait, “Trait → DNAm”(brown square) represents cross-lagged effect from trait to DNA methylation, and “DNAm → Trait” (red triangle) represents cross-lagged effect from DNA methylation to trait

For the 20 overall stroke-related CpGs, we observed 142 significant autoregressive and cross-lagged associations identified from the ALT-SR model, of which 45 were cross-lagged effects of methylation on cardiometabolic traits, and six were effects from traits to methylation. Again, TG, BMI, SBP, and DBP accounted for the majority of the autoregressive associations. The CpGs with the highest number of cross-lagged effects were cg01408932, cg05566961, cg06151165, cg07290552, cg10859726, and cg18146799 predicting SBP and DBP in at least two time points. The latter three CpGs were also found to have cross-lagged effects on BMI. The cross-lagged effect from traits to DNA methylation happened in DBP predicting the methylation level of cg05530317, cg01408932, and cg06151165 at one or more time points, and in SBP at IPT3 determining the methylation level of cg05566961 at IPT5. No big differences for cross-lagged effect were found between all sample and subsamples, and between MZ and DZ (Fig. 2, Additional file 7: Figure S6, Additional file 8: Figure S7, Additional file 1: Table S10A–S10G).

**Fig. 3:**
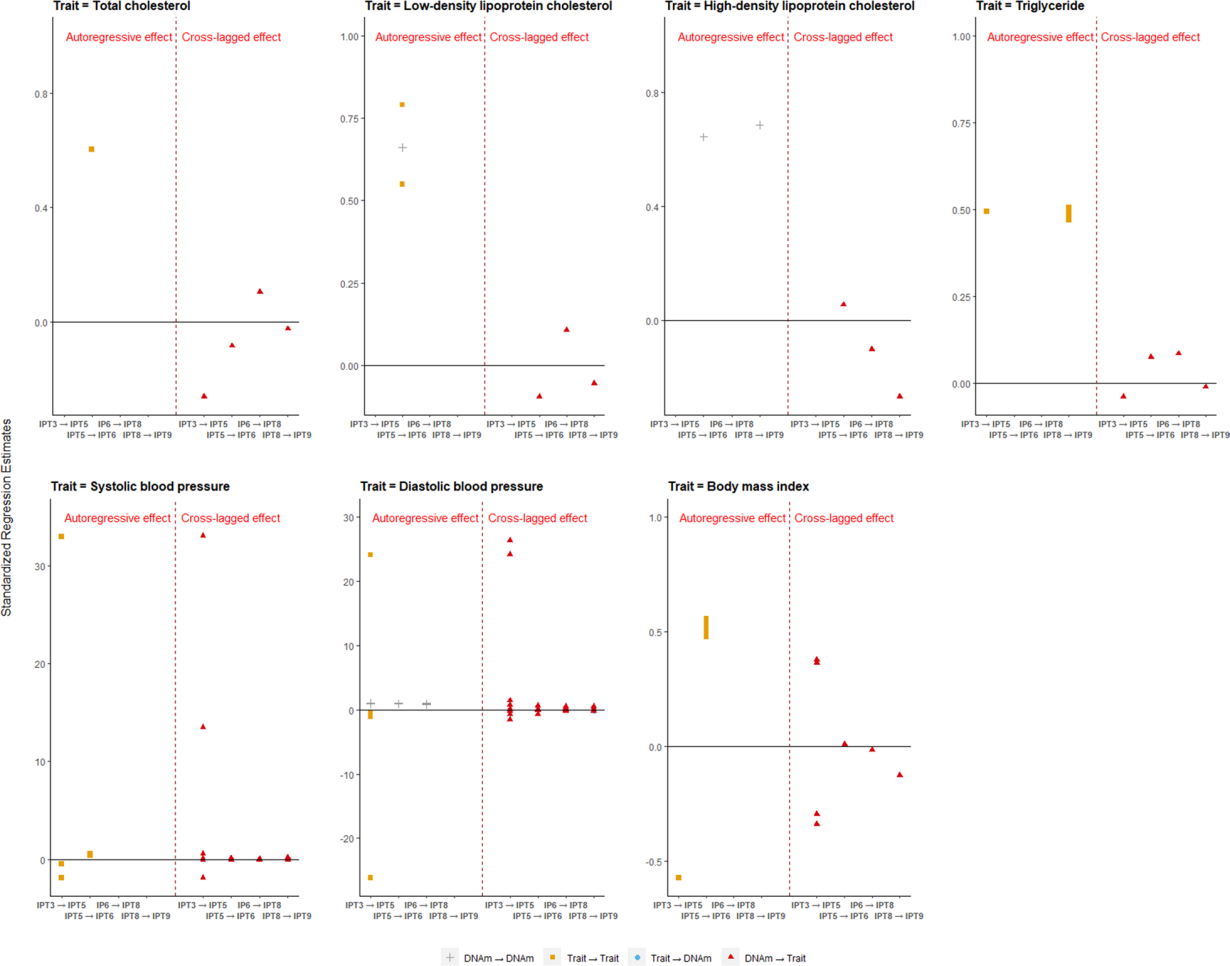
Autoregressive and cross-lagged effect between DNA methylation of ischemic stroke-related CpGs and cardiometabolic traits in all sample. Each point represents one significant effect (*P* value was set to 3 $\times$ 10^–4^). The *X*-axis represents the effect at different adjacent time points; for example, IPT3 → IPT5 means the effect of one variable at IPT3 on the other variable at IPT5. The *Y*-axis represents the standardized estimation coefficient from the regression model. The left part of the figure is the autoregressive effect, and the right part is the cross-lagged effect. “DNAm → DNAm” (gray plus sign) represents autoregressive effect of DNA methylation, “Trait → Trait” (blue circle) represents autoregressive effect of trait, “Trait → DNAm”(brown square) represents cross-lagged effect from trait to DNA methylation, and “DNAm → Trait” (red triangle) represents cross-lagged effect from DNA methylation to trait

The ALT-SR analysis based on ischemic stroke-related CpGs showed that 154 significant associations were composed of 66 cross-lagged effects of DNA methylation on cardiometabolic traits, one cross-lagged effect from methylation on trait, as well as 87 autoregressive effects. DNA methylation level of cg02947021 had multiple time point cross-lagged effects on all cardiometabolic traits except SBP, because the ALT-SR model failed to converge for the analysis of SBP. Moreover, DNA methylation of cg03909417, cg07290552, cg10177207, cg10450108, cg14201424, and cg25889711 showed cross-lagged effects on SBP, DBP, or both at multiple time points. The only significant cross-lagged effect from trait to DNA methylation was found in DBP at IPT8 predicting DNA methylation of cg05530317 at IPT9. No big differences for cross-lagged effect were found among different samples (Fig. 3, Additional file 9: Figure S8, Additional file 10: Figure S9, Additional file 1: Table S11A–S11G).

In order to avoid potential bias caused by the selection of CpGs and to increase the comparability with other studies, we also selected 20 top-ranked CpGs from EWAS analysis specific for these cardiometabolic traits (Additional file 1: Table S12) to do the sensitivity analysis for cross-lagged effect. Sensitivity analysis for the ALT-SR model showed that the autoregressive effect was accounted for by TG, BMI, SBP, and DBP, and the majority of significant cross-lagged paths were in the direction from DNA methylation to traits, which was similar to the findings in the main analysis. However, the majority of significant cross-lagged paths were found in CpGs relating to TC, TG, and SBP, which was not consistent with the findings in the ALT-SR analysis of CVD-related CpGs (Additional file 1: Table S13A–S13G).

### Mediation effects linking DNA methylation, cardiometabolic traits, and CVD

Since we found the most cross-lagged effects in CVD-related CpGs on subsequent cardiometabolic traits, the longitudinal mediation model was established with cardiometabolic traits as the mediation variable in the path between DNA methylation and CVD. We used the same significance level as in the ALT-SR model for multiple testing.

In the mediation analysis for non-stroke CVD, we found 31 associations measuring the direct effects of either DNA methylation or traits on CVD, and 7 measuring indirect effects. Direct effects originating from the slope of DNA methylation accounted for 80% (16/20) of the direct effects of DNA methylation on CVD. Direct effects of traits on CVD were mainly contributed by TC, LDL, BMI, SBP, and DBP. Indirect effects of CpGs to non-stroke CVD were found in cg01417615 and cg20729301 where LDL was treated as the mediator, and in cg20729301 where BMI was the mediator, and also in two CpGs (cg11521799 and cg20921874) where SBP was the mediator. For the converged mediation model, there was some different indirect effect pattern among all sample and subsamples, for example, we found significant mediation effect from cg24610274 to non-stroke CVD mediated by LDL in DZ sample, but not in all sample and MZ sample, and we found significant indirect effect of TC in this CpG site in DZ but not in MZ. (Additional file 11: Figure S10, Additional file 12: Figure S11, Additional file 13: Figure S12, Additional file 1: Table S14A–S14E).

In the mediation analysis with overall stroke as outcome, we found 132 significant paths. Direct effects caused by slope of DNA methylation and slope of traits accounted for about 70% (47/66) and 95% (17/18) of the total direct effects of each construct, respectively. The highest number of indirect effects was identified when LDL and HDL were treated as mediators; for example, LDL had significant mediation effects in the path from multiple CpGs (cg12662929, cg17764549, cg25184724, cg05566961, cg06889348, and cg23250494) to overall stroke, and HDL had significant mediation effects for cg12662929, cg07897701, cg06151165, cg23250494, and cg02449762 on overall stroke. Moreover, TC had mediation effect in the path from cg11653466 to overall stroke and DBP has mediation effect for cg25936482. For the converged model, there was small different indirect effect pattern among all sample and subsamples; for example, DBP has a significant mediating effect in the path from cg11188837 to ischemic stroke in MZ, not in all sample and DZ (Additional file 14: Figure S13, Additional file 15: Figure S14, Additional file 16: Figure S15, Additional file 1: Table S15A–S15F).

Mediation analysis for ischemic stroke revealed a sum of 56 significant paths, consisting of 40 direct associations, six indirect associations, and 10 total effects. Direct associations were contributed by the intercept and slope of DNA methylation, as well as the slope of cardiometabolic traits (TC, LDL, BMI, and SBP). The indirect effect was identified in the path from cg03909417 mediated by TC, from cg22941668 mediated by LDL, from cg10177207 mediated by BMI, and from cg25889711 mediated by SBP. The different indirect effect pattern was a bit different among all sample, MZ and DZ for some CpGs; for example, BMI had significant mediating effect for the association between cg10177207 and ischemic stroke in all sample and MZ sample, but not in DZ sample (Additional file 17: Figure S16, Additional file 18: Figure S17, Additional file 19: Figure S18, Additional file 1: Table S16A–S16F).

## Discussion

In this longitudinal EWAS of CVD in a twin-based population, we identified 20 top-ranked CpGs associated with non-stroke CVD, overall stroke, and ischemic stroke, respectively, where only one ischemic stroke-related CpG site reached Bonferroni-adjusted significance level in DZ. We further assessed cross-lagged effects between these CVD-related CpGs and cardiometabolic traits (blood lipids, blood pressure, and BMI) over two decades, where DNA methylation at these CpGs predicted the level of all cardiometabolic traits at the adjacent follow-up time point, but the other direction was rarely found. In the mediation analysis, in addition to the direct effects from either DNA methylation or cardiometabolic traits on CVD, the indirect effects of some CpGs were mediated by cardiometabolic traits. Sensitivity analysis using different sets of CpGs for cross-lagged effect showed a similar direction but different trait contribution for cross-lagged effects compared to that in the main analysis, demonstrating less bias in the selection of CpGs for data analysis as well as complex associations among DNA methylation, cardiometabolic traits, and CVD.

### EWAS findings in comparison with previous evidence

The top-ranked CpGs identified in our EWAS did not overlap with those of previous EWAS studies on either non-stroke CVD or stroke [4, 5]; however, genes mapped by these CpGs were reported to be associated with CVD or cardiometabolic traits in different ways in previous studies. For example, previous studies showed that genetic variants in *SKI* were related to coronary artery disease [14], and CpGs located in *SKI* were associated with smoking [15]. Genetic variants in *TMUB2* (Transmembrane and ubiquitin like domain containing 2) were associated with multiple measures, such as BMI and body fat distribution [16], and we found that cg04482923 in this gene had a significant cross-lagged effect on BMI. In the gene sets related to overall stroke or ischemic stroke in our study, we identified *ABP1*, also known as *AOC1* (amine oxidase copper containing 1), which has been associated with HDL [17], TG [18], and BMI [19]. We found an indirect effect of cg07897701 in *ABP1* on overall stroke mediated by HDL. Taken together, the CVD-related CpGs identified in our EWAS had prior evidence of importance for CVD risk in these mapped gene regions.

### Co-varying pattern between DNA methylation and cardiometabolic traits

In the ALT-SR analysis using up to 20 years of longitudinal data, we found that during late adulthood, changes in DNA methylation of CVD-related CpGs were likely to predict changes in cardiometabolic traits, but less likely to be reversely predicted by cardiometabolic traits. This pattern was consistent for different CpG sets identified in the EWAS analysis, for different cardiometabolic traits, as well as for different samples (all sample, MZ and DZ). With the number of EWAS studies increasing and a growth in the use of Mendelian randomization (MR), a method for causal inference, a great deal of evidence has been produced for the relationship between DNA methylation and cardiometabolic traits. However, these studies produced mixed results, especially for causal associations.

#### Lipids and DNA methylation

A bidirectional longitudinal association study conducted by Morrison et al. (*n* = 179, 2 years of follow-up time) demonstrated that the cross-lagged effect was only significant between epigenetic age at an earlier time point and metabolic syndrome (or its component, denoted as lipid/obesity) at a later time point [20]. Another study identified 101,911 *cis*-methylation quantitative trait loci (meQTLs, defined as genetic variants-CpG associations within the same gene locus) and 5342 *trans*-meQTLs (within different gene loci) associated with DNA methylation in human adipose tissue. They further reported a mediating role of DNA methylation between genetic variants and metabolic traits such as BMI and lipids with a causal inference test [21]. However, another EWAS study using a stepwise MR analysis in a sample of 3,296 Dutch individuals demonstrated that blood lipids determined the methylation of genes related to lipid metabolism, and not vice versa [22]. Although there were some exceptions, longitudinal studies and MR studies give relatively consistent results with our study, such that DNA methylation potentially predicts lipids levels.

#### BMI and DNA methylation

Previous studies tend to conclude that BMI was the cause of changes in DNA methylation, rather than its consequence. For example, Wahl et al. demonstrated the more likely causal direction from BMI to DNA methylation in an EWAS study (*n* = 10,261) [23]. Another large-sample EWAS study using MR approach (discovery population: 3743; replication population: 4055) identified 83 BMI-related CpGs, of which genetically predicted DNA methylation at one specific site was associated with BMI, and another 16 CpGs were secondary to BMI [24]. The following three studies demonstrated the cross-lagged effect between DNA methylation and BMI. As mentioned above, Morrison et al. showed significant cross-lagged effect of epigenetic age on obesity [20]. Another study found that DNA methylation at follow-up was predicted by baseline BMI 6.2 years prior, and no significant associations were found between baseline DNA methylation and BMI at follow-up [25]. The third study using data from mothers (*n* = 792) and children (*n* = 906) revealed cross-lagged effects between early-life BMI and later-life DNA methylation score in both populations. However, the MR analysis did not find a significant association between genetically predicted BMI and DNA methylation, but only demonstrated a very weak association between meQTLs and BMI [6]. It is worth mentioning that one study based on the same twin sample as our study had similar findings as our study, that is, baseline levels of DNA methylation at five specific CpGs were significantly associated with BMI at follow-up or associated with change in BMI [26]. Evidence of downstream effects of DNA methylation on BMI was also shown in another study identifying 92 causal CpGs for both CVD and its risk factors, four of which were for BMI [27]. Therefore, results are inconsistent, although more studies support the finding that BMI causes DNA methylation than the opposite.

#### BP and DNA methylation

Few studies have investigated the associations between BP and DNA methylation. A cross-sectional designed EWAS study (*n* = 17,010) identified 13 CpGs associated with BP and demonstrated bidirectional associations between two of these CpGs and BP in MR analysis [9]. Another trans-ancestry genome-wide association study showed that genetic variants associated with BP were also associated with DNA methylation at nearby CpGs (meQTL), suggesting a regulatory role of DNA methylation in the pathway linking genetic variants and BP [28]. Morrison et al. did not find a significant cross-lagged effect between epigenetic age and BP [20].

### Role of co-varying patterns of DNA methylation and cardiometabolic traits in CVD

In the mediation analysis, the associations between DNA methylation of CVD-related CpGs and CVD were further strengthened (significant direct effect from DNA methylation on CVD). We also found significant indirect effects in the pathway from DNA methylation to CVD mediated by cardiometabolic traits, although the mediating effect only existed in a few CpGs.

A recent genome-wide module-based epigenetic study of incident CVD conducted by Westerman et al. not only gave similar results as ours regarding the DNA methylation role in the process of CVD, but also suggested possible mechanisms of DNA methylation in CVD during different stages along the entire life course. The authors identified three clusters of DNA methylation data, named brown, blue, and purple modules, to be strongly related to CVD, and the genes in these modules were found to be enriched in the processes of either immune function (brown module) or development (blue module). The brown module tended to have stronger correlations with cardiovascular risk factors, such as BP, lipids, and BMI. Mediation analysis demonstrated that the association between the brown module with CVD was weaker after adjusting for CVD risk factors than the converse, suggesting that the brown methylation module may act as a biomarker, rather than a mediator, for the actions of CVD risk factors [3].

As Westerman and colleagues pointed out, the two modules proposed in the study may represent two different mechanisms of CVD: one is the long-lasting risk of early-life exposure, and the other is the inflammation mechanism during the life course. Interestingly, a similar module pattern was also obtained in an EWAS network analysis on chronic obstructive pulmonary disease [29], another age-related disease. The association between DNA methylation, early-life exposure, and disease in later life has been widely investigated in animal studies [30], famine population studies [31], and parent–offspring studies [6]. These studies support the hypothesis that DNA methylation responds to early-life exposure, such as maternal undernutrition/over-nutrition, stress, and household socioeconomic status [32]. Altered DNA methylation may persist, influence gene expression, and cause transcriptional interference and genomic instability and ultimately influence health in later life [32, 33]. On the other hand, atherosclerosis and inflammation are the primary pathological bases of CVD. Atherosclerosis refers to thickness of the arterial intima and the formation of atherosclerotic lesions and is induced by the complex interplay of various environmental risk factors, together with genetic background and DNA methylation regulation [34]. The relationship between environmental risk factors and DNA methylation becomes more uncertain as age increases. From our results, although we identified a more relevant pattern of cross-lagged effect by DNA methylation predicting major CVD risk factors, we also found that DNA methylation at some CpGs was determined by these risk factors. From the literature we reviewed, the research results appear inconsistent, even with similar study designs, similar measurement techniques for DNA methylation, and similar definitions of phenotypes. The inconsistency may in part be due to population features, sample size, time intervals of repeated measurements, statistical models, measurement error, and unknown mechanisms, which will pose challenges for future research and prevention strategies.

## Strengths and limitations

The strengths of the current study are reflected in several aspects. First, as far as we know, this is the first study to demonstrate cross-lagged effect patterns between DNA methylation and cardiometabolic traits at multiple time points during a long period. The ALT-SR model we used is a well-studied cross-lagged effect model used to separate between- and within-person effects. Second, we used twins in our study, who are naturally matched in many genetic and environmental risk factors and have unique value to study DNA methylation. Moreover, the study population is based on the Swedish Twin Registry (STR) [35], which is the largest nationwide twin registry in the world and has reliable and continuous data resources for research. Therefore, multiple analyses could be performed to evaluate the causal role of DNA methylation in CVD. However, some limitations should be addressed. First, the study was performed in a relatively small sample for both EWAS analysis and ALT-SR analysis. Researchers have suggested that 98 MZ twins are needed to reach a power of 80% in detecting a 10% mean difference of DNA methylation between discordant twins at an EWAS significant threshold of 1 $\times$ 10^–6^ [36]. For the growth model, it was suggested that a sample size of at least 100 is more preferable; however, three or more repeated measurements per person are also recommended to get a well-fitting model [37]. Although the sample size is not very large, the model fit measurements from the ALT-SR analysis (Additional file 1: Table S9A–S11G, Additional file 1: Table S13A–S13G) and consistent findings from our EWAS study compared with previous studies (for example, cg17901584 and cg27243685, which were identified in our EWAS analysis on lipids (Additional file 1: Table S12), were also reported in previous large sample EWAS study [8]) implied a relatively robust and reliable result under the current sample size. In addition, because of the lack of statistical power caused by the small sample size, we did not analyze more detailed CVD categories. Second, the study population is an older cohort with a mean baseline age of 73 years and of European ancestry, so the results may not be generalizable to other populations. Third, a large portion of the CpG sites were excluded from the analyses (only 255,356 CpGs remaining) after the quality control, which could potentially lead to exclusion of regions of relevance in CVD. This limitation might have impacted on the results and conclusions of the research. Finally, results from DNA methylation data could not alone provide a full picture of the biological mechanisms underlying CVD. Therefore, the directions for our future research should be using better coverage data such as that from whole genome bisulfite sequencing [38], and integration of multi-omics data to provide a deeper understanding of the association between different risk factors and CVD.

## Conclusions

The cross-lagged effects and mediation effects discovered in this study provide insights into the complex associations between DNA methylation and CVD. We show that DNA methylation levels of CVD-related CpGs are more likely to determine the levels of cardiometabolic traits, than the opposite. We further discovered that the association between DNA methylation at a few of these CpGs and CVD may be mediated by cardiometabolic traits. Further research is needed to investigate the epigenetic basis of CVD risk as well as the role of DNA methylation as a biomarker of CVD for disease prevention, interventions, and prognosis.

## Methods

### Study population

The study population is based on SATSA, a sub-study of the STR. The study design, population characteristics, and data collection of SATSA can be found in previous publications [12, 39]. Briefly, SATSA collected longitudinal information on aging-related phenotypes in up to ten repeated waves of assessments between 1984 and 2014 in twin pairs in Sweden. Assessment in each wave included a questionnaire survey and an IPT, where the latter was the source of DNA methylation and cardiometabolic traits in this study. Each IPT consisted of a physical examination, structured cognitive tests, and blood sample collection, except for the fourth IPT where only a telephone survey was implemented. Blood samples were used for biochemical testing, genetic, and epigenetic testing.

### Cardiovascular disease ascertainment

Identification of diagnoses of CVD was conducted by linking the Swedish National Patient Register and Causes of Death Register to SATSA through the unique personal identity number (ID) assigned to all Swedish residents, here the follow-up in the registers was throughout 2016. CVD in this study included non-stroke CVD and stroke. Non-stroke CVD was defined as any of the following diagnosis in the two registry databases: angina pectoris, arteriosclerosis, intermittent claudication, ischemic heart disease, myocardial Infarction. Stroke was recorded as overall stroke, which included all types of stroke in the registry database, and was also specified by subtypes including ischemic and hemorrhagic stroke. Previous studies revealed little overlap for DNA methylation in non-stroke CVD and stroke [40]. Moreover, stroke is a heterogeneous disease and ischemic stroke is the main type of overall clinical stroke; therefore, they were analyzed separately. International Classification of Diseases (ICD) codes for the classification of CVD are listed in Additional file 1: Table S17.

### Definition and measurement of cardiometabolic traits and other covariates

Blood lipids, BP, height, and weight were measured in each IPT. BP measurement value (mmHg) after resting for five minutes was used. BMI was calculated as weight (kg) divided by height (meters) squared. Measurement methods or procedures have been described in previous studies [41, 42]. The participants’ smoking status was self-reported at each IPT and was categorized as non-smoker, ex-smoker, and current smoker. The variable of statin use was also derived from self-reported investigation of IPT as well as the Swedish Prescribed Drug Register.

### DNA methylation measurements

Genome-wide DNA methylation was repeatedly measured in participants over different IPTs: IPT3 (1992–1994), IPT5 (1999–2001), IPT6 (2002–2004), IPT8 (2008–2010), IPT9 (2010–2012), and IPT10 (2012–2014). Genomic DNA was isolated from peripheral whole blood samples, and DNA methylation was measured using Infinium HumanMethylation450 BeadChips and the Illumina EPIC Human Methylation Microarray. Methylation data was processed for quality control, normalization and adjustment for cell counts and batch effects, and details can be found in previous studies [39]. Briefly, quality control was conducted to filter out CpGs and samples if: (1) the probes had a detection *p* value greater than 0.05; (2) the probes had SNPs overlapping the CpG site; (3) samples had mislabeled gender based on sex chromosome data; and (4) samples had poor correlation with genotyping information. CpGs present and passing quality control on both arrays were included in the analysis, leaving 255,356 CpGs for the analysis. After background correction and normalization of the raw data, cell counts and batch effect-adjusted methylation beta values were used in the data analysis.

### Statistical analysis

**Fig. 4:**
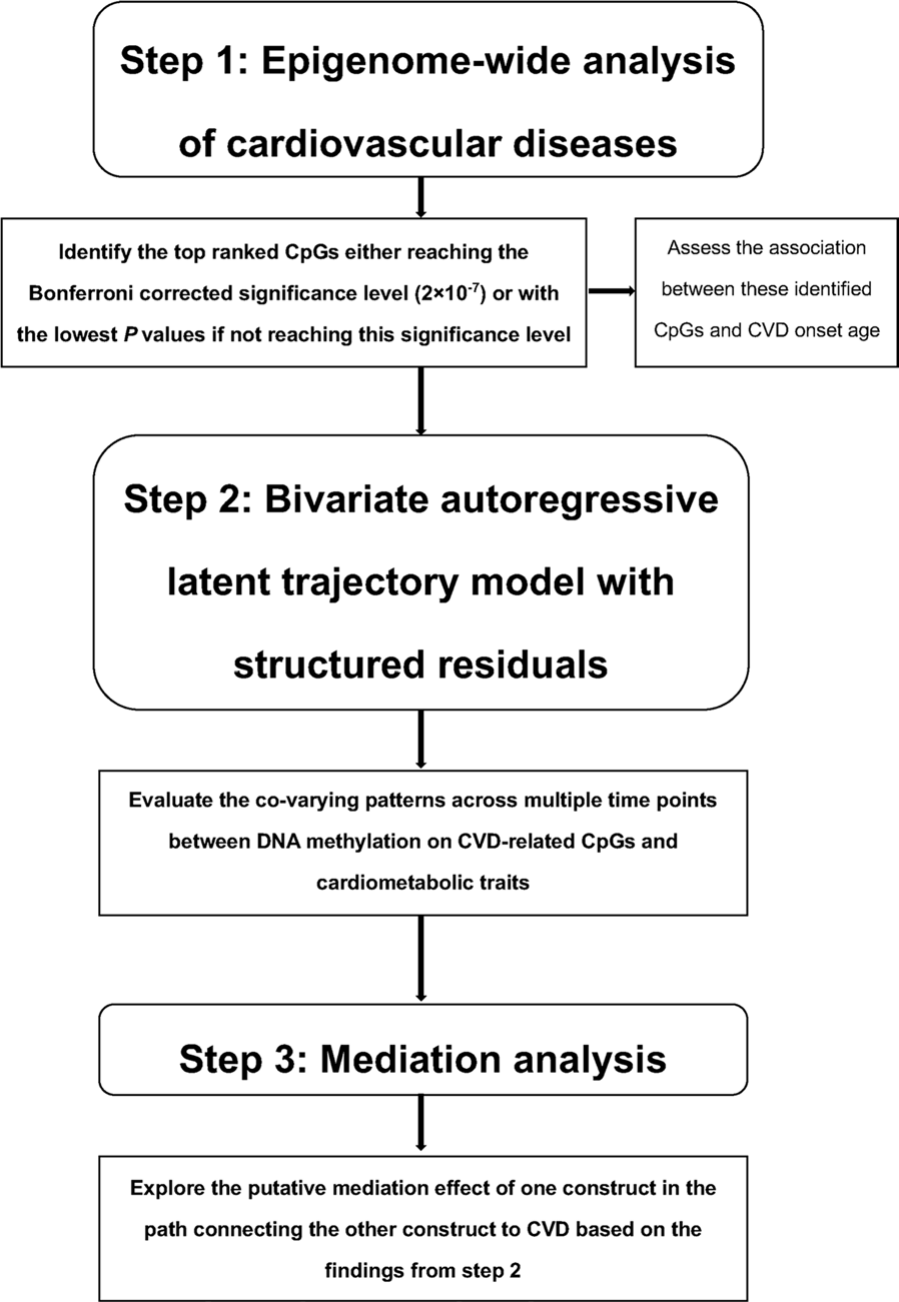
Flow chart of the statistical analyses. *CVD* cardiovascular diseases. Rounded rectangle represents the statistical steps, and right-angled rectangle represents the statistical contents under each step

In participants diagnosed with CVD, if IPT date happened earlier than CVD diagnosed date, the diagnosis of CVD was coded as 0 (no CVD), otherwise coded as 1 (had CVD). Formula “LDL = TC – HDL − TG/5” was used to impute the missing values of LDL. Statin use was coded as 1 if the participant was recorded using statin at least once, otherwise coded as 0. The statistical analysis was performed in three steps (Fig. 4).

### Step 1: EWAS of CVD

A linear mixed-effect model was used to test the association, where DNA methylation level of each CpG site was entered as a dependent variable, CVD was independent variable with a fixed effect, personal ID nested within twin ID was entered as a random effect. and personal ID nested within twin ID was entered as a random effect. Repeated measured age (continuous variable), sex (binary variable, female was coded as 1, male was coded as 0) and repeated measured smoking (non-smoker was coded as 1, ex-smoker was coded as 2, and current smoker was coded as 3) were covariates as fixed effects. The epigenome-wide significance threshold was Bonferroni-corrected and set to *P* value less than 2 × 10^–7^. CpGs were selected for the following analysis if they reached this significance level or were among the top 20 ranked CpGs with the lowest *P* values if they did not reach the significance level. We further assessed the association between DNA methylation level of CVD-related CpGs and CVD onset using two methods, case-only analysis and survival analysis. In case-only analysis, we restricted the analytic sample to those who had been diagnosed with CVD after any of their IPT dates. We then fitted a linear mixed model with the onset age of CVD as dependent variable, methylation level of CVD-related CpGs as independent variable with fixed effect, and twin ID as the random effect. Since some participants may have several measurements of DNA methylation eligible for this analysis, we chose the first measurement, last, random selected and the mean levels of DNA methylation, to fit the model separately. Covariates were chosen and entered into the model in the same way as in the EWAS step. In the survival analysis, baseline was the first IPT measurement of DNA methylation and participants were excluded if they had the diagnosis of CVD before baseline. End of follow-up was defined by the diagnosis date or December 31, 2016, which was the last date of CVD diagnosis in the registry database. Participants who died without any CVD diagnosis or survived until the last date of CVD diagnosis were censored. Attained age was used as the underlying time scale. Definition of independent variable and covariates (sex and smoking status) were the same as in the above case-only analysis.

### Step 2: ALT-SR

**Fig. 5:**
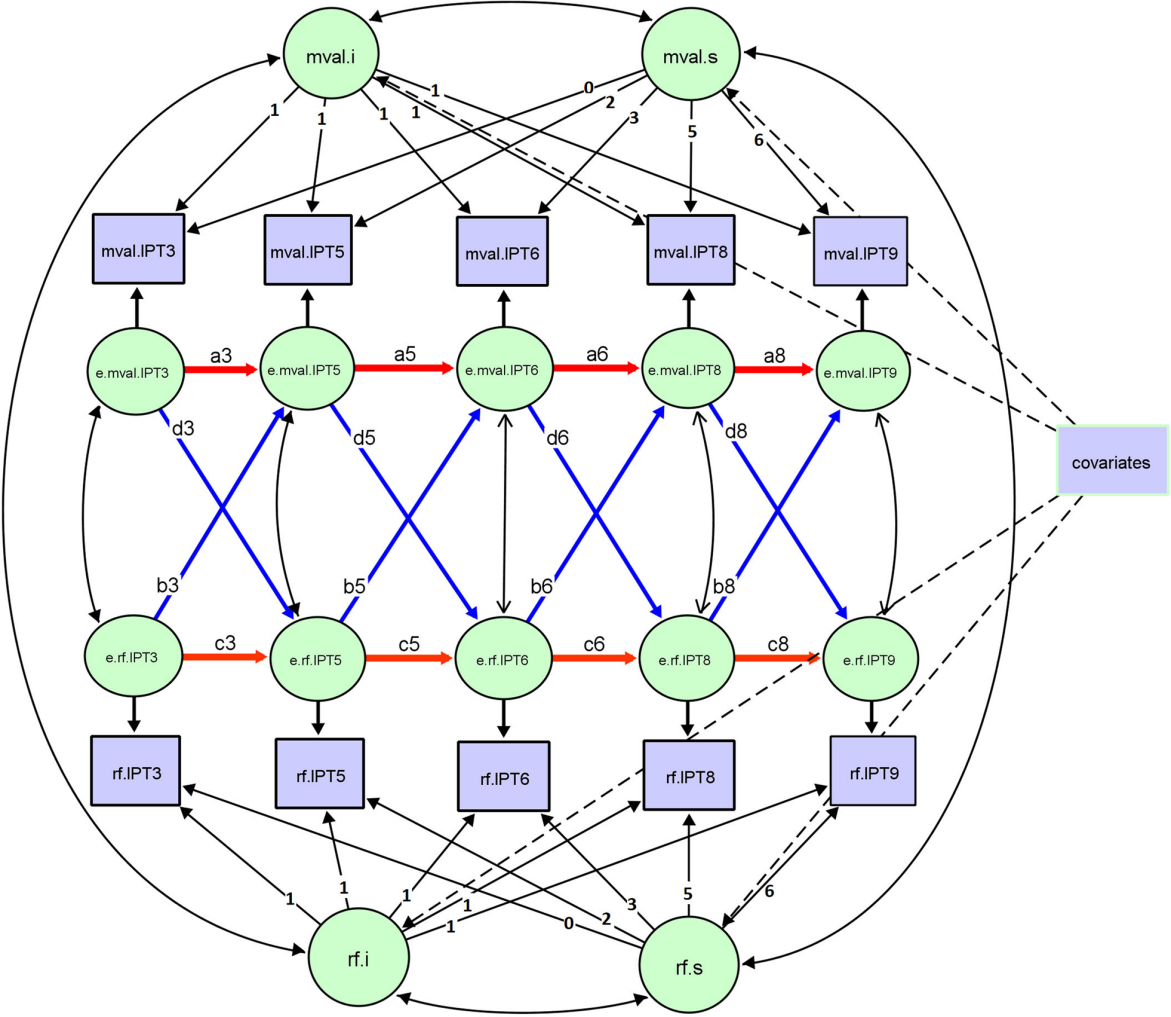
Bivariate autoregressive latent trajectory model with structured residuals. This figure illustrates a bivariate autoregressive latent trajectory model with structured residuals (ALT-SR). The manifest variables (observed in the study) are denoted as rectangles, and the latent variables (not observed) are denoted as circles, double headed arrows are the variance or covariance of variables, single headed arrows are either factor loadings (from latent variables to manifest variable) or regressions. “mval” is the abbreviation for methylation value and “rf” is the abbreviation for risk factor. So mval.IPT3 to mval.IPT9 represents the observed level of DNA methylation at different time points for one specific CpG site, and rf.IPT3 to rf.IPT9 represents the observed level of one specific cardiometabolic trait at different time points. ALT-SR comprised two parts, the autoregressive model (AR) and the latent growth curve (LGM), where LGM is initially modeled, followed by AR analysis. In LGM, the expected trajectory is established by two latent variables, the intercept (denoted as mval.i and rf.i) measuring the individual baseline levels of DNA methylation and cardiometabolic traits, respectively, and the slope (denoted as mval.s and rf.s) measuring the changing rate over time for every person. Since we fit a linear growth model for both DNA methylation and cardiometabolic traits, the factor loadings from latent intercept to manifest variables at different time points are all equal to 1, and the factor loadings from latent slope to manifest variables are set to 0, 2, 3, 5 and 6 at IPT3, IPT5, IPT6, IPT8, and IPT9, respectively. The AR part has two sets of regression paths based on the latent residuals of DNA methylation and the specific cardiometabolic trait, respectively: the autoregressive path (e.g., e.mval.IPT3 → e.mval.IPT5 → e.mval.IPT6 → e.mval.IPT8 → e.mval.IPT9) indicating the within-person changes of each of the two constructs over time, and the cross-lagged path (e.g., e.mval.IPT3 → e.rf.IPT5, e.rf.IPT3 → e.mval.IPT5) indicating whether DNA methylation at one time point predicts within-person changes of cardiometabolic traits at an adjacent later time point, and/or vice versa. Therefore, a3–a8 represent the autoregressive parameters of DNA methylation, c3–c8 represent the autoregressive parameters of cardiometabolic traits, b3–b8 represent the parameters of cross-lagged effect of cardiometabolic traits on DNA methylation, and d3–d8 represent the parameters of cross-lagged effect from DNA methylation to cardiometabolic traits. The variance and covariance of the latent residuals of DNA methylation and cardiometabolic traits are constrained to be equal across time except for the first occasion. We include time-independent covariates that influence the growth curve estimates; here sex and baseline age are common covariates when fitting the ALT-SR for different CpGs and cardiometabolic traits and include statin use as another time-independent variable when fitting the model for lipids. The relatedness of twins in the model is also adjusted for. Variance of mval.i, mval.s, rf.i, rf.s, e.mval.IPT3-e.mval.IPT9, e.rf.IPT3-e.rf.IPT9 is not displayed in the figure

The ALT-SR model has the advantage, compared to a traditional cross-lagged model, to separate the between-person and within-person associations between constructs and therefore allow us to estimate the directional relations between DNA methylation and cardiometabolic traits over time from the cross-lagged parameters. The fitted model is shown in Fig. 5. As defined in a standard structural equation model, the manifest variables (observed in the study) are in the rectangle, and the latent variables (not observed) are in the circle, double head arrows are the variance or covariance of variables, single head arrows are either factor loading (from latent variables to manifest variable) or regressions. “mval” is the abbreviation for DNA methylation and “rf” is the abbreviation of risk factor, representing cardiometabolic traits; therefore, mval.IPT3 to mval.IPT9 mean the observed level of DNA methylation at different time points for one specific CpG site, and rf.IPT3 to rf.IPT9 represent the observed level of one specific cardiometabolic trait at different time points. The ALT-SR model comprised two parts, the autoregressive model (AR) and the latent growth curve model (LGM), where LGM was initially modeled, followed by AR analysis. In LGM, the expected trajectory was established by two latent variables: the intercept measuring the individual baseline level (for example, mval.i and rf.i mean the baseline levels of DNA methylation and cardiometabolic traits, respectively) and the slope (denoted as mval.s and rf.s) measuring the changing rate over time for each person. Since we fit a linear growth model for both DNA methylation and cardiometabolic traits, the factor loading from latent intercept to manifest variable at different time points is all equal to 1, and the factor loading from latent slope to manifest variables is set as 0, 2, 3, 5, and 6 at IPT3, IPT5, IPT6, IPT8, and IPT9, respectively. In AR, there are two sets of regression paths based on the latent residuals of two constructs (DNA methylation of one specific CpG site and one specific cardiometabolic trait): autoregressive path (e.g., e.mval.IPT3 → e.mval.IPT5 → e.mval.IPT6 → e.mval.IPT8 → e.mval.IPT9) indicating the within-person changes of each of the two constructs over time, and cross-lagged path (e.g., e.mval.IPT3 → e.rf.IPT5, e.rf.IPT3 → e.mval.IPT5) indicating whether DNA methylation at one time point predicts within-person changes of cardiometabolic traits at the adjacent later time point, and/or vice versa. Therefore, a3–a8 are labeled as the autoregressive parameters of DNA methylation, c3–c8 are labeled as the autoregressive parameters of cardiometabolic traits, b3–b8 are labeled as the parameters of cross-lagged effect of cardiometabolic traits on DNA methylation, and d3–d8 are labeled as parameters of cross-lagged effect from DNA methylation to cardiometabolic traits. We constrained the variance and covariance of the latent residuals of DNA methylation and cardiometabolic traits to be equal across time except for the first occasion, but let the autoregressive and cross-lagged associations to be freely estimated over time. We also included time-independent covariates that influence the growth curve estimates, here sex and baseline age were the common covariates when fitting the ALT-SR for different CpGs and different cardiometabolic traits, and we also included statin use when fitting the model for lipids. We used chi-square value (relevant *P* value > 0.05), root-mean-square error of approximation (RMSEA) less than 0.06, comparative fit index (CFI) larger than 0.95 as the threshold to suggest a well-fit model [43]. The relatedness of twins was adjusted in the model by using the cluster option in the Lavaan package in R. Moreover, DNA methylation data scared in IPT10, HDL, and LDL data were missing in IPT3 (Additional file 1: Table S1); we therefore fitted the ALT-SR model for TC, TG, SBP, DBP, and BMI at 5 time points (IPT3, IPT5, IPT6, IPT8, IPT9) and fit the model for HDL and LDL at 4 time points (IPT5, IPT6, IPT8, and IPT9) in model convergence. Rescaling was only performed for methylation data by multiplying the raw DNA methylation data by 10 in order to get a converged model.

In order to avoid potential bias caused by the selection of CpGs, we also selected the top-ranked CpGs from EWAS analysis specific for these cardiometabolic traits to do the sensitivity analysis for cross-lagged effect. Similarly, we performed EWAS analysis on cardiometabolic traits separately and then fit the ALT-SR model to assess the cross-lagged effect between specific trait-related CpGs and that specific trait. Except the specific cardiometabolic trait was entered as independent variable with fixed effect in mixed model of EWAS analysis, the other settings for both EWAS and ALT-SR analysis, the criterion of selecting CpGs, and the process of calculation were the same as the analyses for CVD.

### Step 3: Mediation analysis

Longitudinal mediation analysis based on findings from the LGM framework was conducted to explore the putative mediation effect of one construct in the path connecting the other construct to CVD. Analysis was restricted to observations where IPT date of measurements happened earlier than the diagnosed date of CVD. A mediation path was established in the framework of LGM, that is DNA methylation and trait grew by time, the random intercept of two constructs’ growth model correlated with each other and then based on the findings from ALT-SR, for example (Additional file 20: Figure S19), we assumed that we found DNA methylation has cross-lagged effect on cardiometabolic traits; mediation path would be established connecting DNA methylation (either intercept or slope of DNA methylation growth curve) to CVD with the slope of cardiometabolic traits as mediators. Therefore, × 1 and × 2 are the direct effects from the intercept (representing baseline level) and the slope (representing the growth rate) of DNA methylation growth model of one specific CpG to CVD, respectively. × 3 and × 4 are the direct effects from the intercept and the slope of one specific cardiometabolic trait growth model to CVD, respectively. × 4 * *m*1 represents the indirect path from the baseline level (intercept of the growth model) of DNA methylation to CVD mediated by the trait, × 4 * *m*2 represents the indirect path from the growth (slope of growth model) of DNA methylation to CVD mediated by the trait. Total effect of one path was the sum of direct effect and indirect effect on that path. Definitions of manifest variables, latent intercept and latent slope, covariates, and abbreviations were set the same as in ALT-SR model. Rescaling was performed for the raw DNA methylation data in the same way as in the second step.

All analyses were conducted separately in non-stroke CVD, overall stroke, and ischemic stroke. Bonferroni correction was applied in all the analyses to adjust the significance level for multiple comparisons. All analyses were performed with R software (4.0.3); specifically, we used the Lavaan package (0.6–7) for the ALT-SR and mediation analysis, and we use Ωnyx to create ALT-SR path diagram in Fig. 5 and mediation path in Additional file 20: Figure S19 [44].

## Supplementary Information

**Additional file 1**. Supplementary tables.

**Additional file 2: Figure S1**. Manhattan plot of EWAS study on non-stroke CVD. Model was adjusted for age, sex and smoking status.

**Additional file 3: Figure S2**. Manhattan plot of EWAS study on overall stroke. Model was adjusted for age, sex and smoking status.

**Additional file 4: Figure S3**. Manhattan plot of EWAS study on ischemic stroke. Model was adjusted for age, sex and smoking status.

**Additional file 5: Figure S4**. Autoregressive and cross-lagged effect between DNA methylation of non-stroke CVD-related CpGs and cardiometabolic traits in MZ sample. Each point represents one significant effect (*P* value was set to 3×10^−4^). The X-axis represents the effect at different adjacent time points, for example, IPT3→IPT5 means the effect of one variable at IPT3 on the other variable at IPT5. The Y-axis represents the standardized estimation coefficient from the regression model. The left part of the figure is the autoregressive effect, and the right part is the cross-lagged effect. “DNAm→DNAm” (gray plus sign) represents autoregressive effect of DNA methylation, “Trait→Trait”(blue circle) represents autoregressive effect of trait, “Trait→DNAm”(brown square) represents cross-lagged effect from trait to DNA methylation, and “DNAm→Trait” (red triangle) represents cross-lagged effect from DNA methylation to trait. MZ, Monozygotic twins; DZ, Dizygotic twins.

**Additional file 6: Figure S5**. Autoregressive and cross-lagged effect between DNA methylation of non-stroke CVD-related CpGs and cardiometabolic traits in DZ sample. Each point represents one significant effect (*P* value was set to 3×10^−4^). The X-axis represents the effect at different adjacent time points, for example, IPT3→IPT5 means the effect of one variable at IPT3 on the other variable at IPT5. The Y-axis represents the standardized estimation coefficient from the regression model. The left part of the figure is the autoregressive effect, and the right part is the cross-lagged effect. “DNAm→DNAm” (gray plus sign) represents autoregressive effect of DNA methylation, “Trait→Trait”(blue circle) represents autoregressive effect of trait, “Trait→DNAm”(brown square) represents cross-lagged effect from trait to DNA methylation, and “DNAm→Trait” (red triangle) represents cross-lagged effect from DNA methylation to trait. MZ, Monozygotic twins; DZ, Dizygotic twins.

**Additional file 7: Figure S6**. Autoregressive and cross-lagged effect between DNA methylation of overall stroke-related CpGs and cardiometabolic traits in MZ sample. Each point represents one significant effect (*P* value was set to 3×10^−4^). The X-axis represents the effect at different adjacent time points, for example, IPT3→IPT5 means the effect of one variable at IPT3 on the other variable at IPT5. The Y-axis represents the standardized estimation coefficient from the regression model. The left part of the figure is the autoregressive effect, and the right part is the cross-lagged effect. “DNAm→DNAm” (gray plus sign) represents autoregressive effect of DNA methylation, “Trait→Trait” (blue circle) represents autoregressive effect of trait, “Trait→DNAm” (brown square) represents cross-lagged effect from trait to DNA methylation, and “DNAm→Trait” (red triangle) represents cross-lagged effect from DNA methylation to trait. MZ, Monozygotic twins; DZ, Dizygotic twins.

**Additional file 8: Figure S7**. Autoregressive and cross-lagged effect between DNA methylation of overall stroke-related CpGs and cardiometabolic traits in DZ sample. Each point represents one significant effect (*P* value was set to 3×10^−4^). The X-axis represents the effect at different adjacent time points, for example, IPT3→IPT5 means the effect of one variable at IPT3 on the other variable at IPT5. The Y-axis represents the standardized estimation coefficient from the regression model. The left part of the figure is the autoregressive effect, and the right part is the cross-lagged effect. “DNAm→DNAm” (gray plus sign) represents autoregressive effect of DNA methylation, “Trait→Trait” (blue circle) represents autoregressive effect of trait, “Trait→DNAm” (brown square) represents cross-lagged effect from trait to DNA methylation, and “DNAm→Trait” (red triangle) represents cross-lagged effect from DNA methylation to trait. MZ, Monozygotic twins; DZ, Dizygotic twins.

**Additional file 9: Figure S8**. Autoregressive and cross-lagged effect between DNA methylation of ischemic stroke-related CpGs and cardiometabolic traits in MZ sample. Each point represents one significant effect (*P* value was set to 3×10^−4^). The X-axis represents the effect at different adjacent time points, for example, IPT3→IPT5 means the effect of one variable at IPT3 on the other variable at IPT5. The Y-axis represents the standardized estimation coefficient from the regression model. The left part of the figure is the autoregressive effect, and the right part is the cross-lagged effect. “DNAm→DNAm” (gray plus sign) represents autoregressive effect of DNA methylation, “Trait→Trait” (blue circle) represents autoregressive effect of trait, “Trait→DNAm” (brown square) represents cross-lagged effect from trait to DNA methylation, and “DNAm→Trait” (red triangle) represents cross-lagged effect from DNA methylation to trait. MZ, Monozygotic twins; DZ, Dizygotic twins.

**Additional file 10: Figure S9**. Autoregressive and cross-lagged effect between DNA methylation of ischemic stroke-related CpGs and cardiometabolic traits in DZ sample. Each point represents one significant effect (*P* value was set to 3×10^−4^). The X-axis represents the effect at different adjacent time points, for example, IPT3→IPT5 means the effect of one variable at IPT3 on the other variable at IPT5. The Y-axis represents the standardized estimation coefficient from the regression model. The left part of the figure is the autoregressive effect, and the right part is the cross-lagged effect. “DNAm→DNAm” (gray plus sign) represents autoregressive effect of DNA methylation, “Trait→Trait” (blue circle) represents autoregressive effect of trait, “Trait→DNAm” (brown square) represents cross-lagged effect from trait to DNA methylation, and “DNAm→Trait” (red triangle) represents cross-lagged effect from DNA methylation to trait. MZ, Monozygotic twins; DZ, Dizygotic twins.

**Additional file 11: Figure S10**. Mediation effect between DNA methylation and non-stroke CVD mediated by cardiometabolic traits in all sample. Each point represents one significant effect (*P* value was set to 3×10^−4^ for multiple testing) identified from mediation analysis among one specific CpG site, one specific cardiometabolic trait, and one specific outcome. The x axis represents the categories of direct effect, indirect effect and total effect, and the y axis represents the estimates of the three effects. "x1” (red) and “x2” (purple) represent direct effect from the intercept and the slope of DNA methylation at one specific CpG to CVD, respectively. “x3” (gold) and “x4” (orange) represent the direct effect from the intercept and the slope of one specific trait to CVD, respectively. “mirs” (x4*m1 in the mediation model, green) represents the indirect effect from the intercept of DNA methylation at one specific CpG to CVD mediated by one specific trait. “msrs” (x4*m2 in the mediation model, pink) represents the indirect effect from the slope of DNA methylation at one specific CpG to CVD mediated by one specific trait. “total_mi” (brown) represents the total effect from the intercept of DNA methylation at one specific CpG to CVD and equals to “x1+x4*m1”. “total_ms” (blue) represents the total effect from the slope of DNA methylation at one specific CpG to CVD and equals to “x2+x4*m2”.

**Additional file 12: Figure S11**. Mediation effect between DNA methylation and non-stroke CVD mediated by cardiometabolic traits in MZ sample. Each point represents one significant effect (*P* value was set to 3×10^−4^ for multiple testing) identified from mediation analysis among one specific CpG site, one specific cardiometabolic trait, and one specific outcome. The x axis represents the categories of direct effect, indirect effect and total effect, and the y axis represents the estimates of the three effects. "x1” (red) and “x2” (purple) represent direct effect from the intercept and the slope of DNA methylation at one specific CpG to CVD, respectively. “x3” (gold) and “x4” (orange) represent the direct effect from the intercept and the slope of one specific trait to CVD, respectively. “mirs” (x4*m1 in the mediation model, green) represents the indirect effect from the intercept of DNA methylation at one specific CpG to CVD mediated by one specific trait. “msrs” (x4*m2 in the mediation model, pink) represents the indirect effect from the slope of DNA methylation at one specific CpG to CVD mediated by one specific trait. “total_mi” (brown) represents the total effect from the intercept of DNA methylation at one specific CpG to CVD and equals to “x1+x4*m1”. “total_ms” (blue) represents the total effect from the slope of DNA methylation at one specific CpG to CVD and equals to “x2+x4*m2”. MZ, Monozygotic twins; DZ, Dizygotic twins.

**Additional file 13: Figure S12**. Mediation effect between DNA methylation and non-stroke CVD mediated by cardiometabolic traits in DZ sample. Each point represents one significant effect (*P* value was set to 3×10^−4^ for multiple testing) identified from mediation analysis among one specific CpG site, one specific cardiometabolic trait, and one specific outcome. The x axis represents the categories of direct effect, indirect effect and total effect, and the y axis represents the estimates of the three effects. "x1” (red) and “x2” (purple) represent direct effect from the intercept and the slope of DNA methylation at one specific CpG to CVD, respectively. “x3” (gold) and “x4” (orange) represent the direct effect from the intercept and the slope of one specific trait to CVD, respectively. “mirs” (x4*m1 in the mediation model, green) represents the indirect effect from the intercept of DNA methylation at one specific CpG to CVD mediated by one specific trait. “msrs” (x4*m2 in the mediation model, pink) represents the indirect effect from the slope of DNA methylation at one specific CpG to CVD mediated by one specific trait. “total_mi” (brown) represents the total effect from the intercept of DNA methylation at one specific CpG to CVD and equals to “x1+x4*m1”. “total_ms” (blue) represents the total effect from the slope of DNA methylation at one specific CpG to CVD and equals to “x2+x4*m2”. MZ, Monozygotic twins; DZ, Dizygotic twins.

**Additional file 14: Figure S13**. Mediation effect between DNA methylation and overall stroke mediated by cardiometabolic traits in all sample. Each point represents one significant effect (*P* value was set to 3×10^−4^ for multiple testing) identified from mediation analysis among one specific CpG site, one specific cardiometabolic trait, and one specific outcome. The x axis represents the categories of direct effect, indirect effect and total effect, and the y axis represents the estimates of the three effects. "x1” (red) and “x2” (purple) represent direct effect from the intercept and the slope of DNA methylation at one specific CpG to CVD, respectively. “x3” (gold) and “x4” (orange) represent the direct effect from the intercept and the slope of one specific trait to CVD, respectively. “mirs” (x4*m1 in the mediation model, green) represents the indirect effect from the intercept of DNA methylation at one specific CpG to CVD mediated by one specific trait. “msrs” (x4*m2 in the mediation model, pink) represents the indirect effect from the slope of DNA methylation at one specific CpG to CVD mediated by one specific trait. “total_mi” (brown) represents the total effect from the intercept of DNA methylation at one specific CpG to CVD and equals to “x1+x4*m1”. “total_ms” (blue) represents the total effect from the slope of DNA methylation at one specific CpG to CVD and equals to “x2+x4*m2”.

**Additional file 15: Figure S14**. Mediation effect between DNA methylation and overall stroke mediated by cardiometabolic traits in MZ sample. Each point represents one significant effect (*P* value was set to 3×10^−4^ for multiple testing) identified from mediation analysis among one specific CpG site, one specific cardiometabolic trait, and one specific outcome. The x axis represents the categories of direct effect, indirect effect and total effect, and the y axis represents the estimates of the three effects. "x1” (red) and “x2” (purple) represent direct effect from the intercept and the slope of DNA methylation at one specific CpG to CVD, respectively. “x3” (gold) and “x4” (orange) represent the direct effect from the intercept and the slope of one specific trait to CVD, respectively. “mirs” (x4*m1 in the mediation model, green) represents the indirect effect from the intercept of DNA methylation at one specific CpG to CVD mediated by one specific trait. “msrs” (x4*m2 in the mediation model, pink) represents the indirect effect from the slope of DNA methylation at one specific CpG to CVD mediated by one specific trait. “total_mi” (brown) represents the total effect from the intercept of DNA methylation at one specific CpG to CVD and equals to “x1+x4*m1”. “total_ms” (blue) represents the total effect from the slope of DNA methylation at one specific CpG to CVD and equals to “x2+x4*m2”. MZ, Monozygotic twins; DZ, Dizygotic twins.

**Additional file 16: Figure S15**. Mediation effect between DNA methylation and overall stroke mediated by cardiometabolic traits in DZ sample. Each point represents one significant effect (*P* value was set to 3×10^−4^ for multiple testing) identified from mediation analysis among one specific CpG site, one specific cardiometabolic trait, and one specific outcome. The x axis represents the categories of direct effect, indirect effect and total effect, and the y axis represents the estimates of the three effects. "x1” (red) and “x2” (purple) represent direct effect from the intercept and the slope of DNA methylation at one specific CpG to CVD, respectively. “x3” (gold) and “x4” (orange) represent the direct effect from the intercept and the slope of one specific trait to CVD, respectively. “mirs” (x4*m1 in the mediation model, green) represents the indirect effect from the intercept of DNA methylation at one specific CpG to CVD mediated by one specific trait. “msrs” (x4*m2 in the mediation model, pink) represents the indirect effect from the slope of DNA methylation at one specific CpG to CVD mediated by one specific trait. “total_mi” (brown) represents the total effect from the intercept of DNA methylation at one specific CpG to CVD and equals to “x1+x4*m1”. “total_ms” (blue) represents the total effect from the slope of DNA methylation at one specific CpG to CVD and equals to “x2+x4*m2”. MZ, Monozygotic twins; DZ, Dizygotic twins.

**Additional file 17: Figure S16**. Mediation effect between DNA methylation and ischemic stroke mediated by cardiometabolic traits in all sample. Each point represents one significant effect (*P* value was set to 3×10^−4^ for multiple testing) identified from mediation analysis among one specific CpG site, one specific cardiometabolic trait, and one specific outcome. The x axis represents the categories of direct effect, indirect effect and total effect, and the y axis represents the estimates of the three effects. "x1” (red) and “x2” (purple) represent direct effect from the intercept and the slope of DNA methylation at one specific CpG to CVD, respectively. “x3” (gold) and “x4” (orange) represent the direct effect from the intercept and the slope of one specific trait to CVD, respectively. “mirs” (x4*m1 in the mediation model, green) represents the indirect effect from the intercept of DNA methylation at one specific CpG to CVD mediated by one specific trait. “msrs” (x4*m2 in the mediation model, pink) represents the indirect effect from the slope of DNA methylation at one specific CpG to CVD mediated by one specific trait. “total_mi” (brown) represents the total effect from the intercept of DNA methylation at one specific CpG to CVD and equals to “x1+x4*m1”. “total_ms” (blue) represents the total effect from the slope of DNA methylation at one specific CpG to CVD and equals to “x2+x4*m2”.

**Additional file 18: Figure S17**. Mediation effect between DNA methylation and ischemic stroke mediated by cardiometabolic traits in MZ sample. Each point represents one significant effect (*P* value was set to 3×10^−4^ for multiple testing) identified from mediation analysis among one specific CpG site, one specific cardiometabolic trait, and one specific outcome. The x axis represents the categories of direct effect, indirect effect and total effect, and the y axis represents the estimates of the three effects. "x1” (red) and “x2” (purple) represent direct effect from the intercept and the slope of DNA methylation at one specific CpG to CVD, respectively. “x3” (gold) and “x4” (orange) represent the direct effect from the intercept and the slope of one specific trait to CVD, respectively. “mirs” (x4*m1 in the mediation model, green) represents the indirect effect from the intercept of DNA methylation at one specific CpG to CVD mediated by one specific trait. “msrs” (x4*m2 in the mediation model, pink) represents the indirect effect from the slope of DNA methylation at one specific CpG to CVD mediated by one specific trait. “total_mi” (brown) represents the total effect from the intercept of DNA methylation at one specific CpG to CVD and equals to “x1+x4*m1”. “total_ms” (blue) represents the total effect from the slope of DNA methylation at one specific CpG to CVD and equals to “x2+x4*m2”. MZ, Monozygotic twins; DZ, Dizygotic twins.

**Additional file 19: Figure S18**. Mediation effect between DNA methylation and ischemic stroke mediated by cardiometabolic traits in DZ sample. Each point represents one significant effect (*P* value was set to 3×10^−4^ for multiple testing) identified from mediation analysis among one specific CpG site, one specific cardiometabolic trait, and one specific outcome. The x axis represents the categories of direct effect, indirect effect and total effect, and the y axis represents the estimates of the three effects. "x1” (red) and “x2” (purple) represent direct effect from the intercept and the slope of DNA methylation at one specific CpG to CVD, respectively. “x3” (gold) and “x4” (orange) represent the direct effect from the intercept and the slope of one specific trait to CVD, respectively. “mirs” (x4*m1 in the mediation model, green) represents the indirect effect from the intercept of DNA methylation at one specific CpG to CVD mediated by one specific trait. “msrs” (x4*m2 in the mediation model, pink) represents the indirect effect from the slope of DNA methylation at one specific CpG to CVD mediated by one specific trait. “total_mi” (brown) represents the total effect from the intercept of DNA methylation at one specific CpG to CVD and equals to “x1+x4*m1”. “total_ms” (blue) represents the total effect from the slope of DNA methylation at one specific CpG to CVD and equals to “x2+x4*m2”. MZ, Monozygotic twins; DZ, Dizygotic twins.

**Additional file 20: Figure S19**. Example of Mediation model. This figure demonstrates an example of the mediation path assuming that the effect of DNA methylation on CVD (outcome) is mediated by one specific cardiometabolic trait. A mediation path is established in the framework of LGM, that is DNA methylation and trait grow by time, the random intercept of the two constructs correlate with each other, the mediation path is established connecting DNA methylation (either intercept or slope of DNA methylation growth curve) to CVD with the slope of cardiometabolic traits as mediators. Besides of the mediation path indicating the indirect effect of one construct on the outcome mediated by the other construct, the direct effects path is established from the parameters of LGM of either construct to outcome. We include sex and baseline age as the common time-independent covariates that influence the growth curve estimates in the analysis for all the cardiometabolic traits, and include statin use when fitting the model for lipids. The manifest variables (observed in the study) are in the rectangle, and the latent variables (not observed) are in the circle, double head arrows are variance or covariance of variables, single head arrows are either factor loading (from latent variable to manifest variable) or regressions. “mval” is the abbreviation for methylation value and “rf” is the abbreviation of risk factor. mval.IPT3 to mval.IPT9 mean the observed level of DNA methylation at different time points for one specific CpG site, and rf.IPT3 to rf.IPT9 represent the observed level of one specific cardiometabolic trait at different time points. mval.i and mval.s represent the intercept and slope of the growth model of DNA methylation on one specific CpG site, rf.i and rf.s represent the intercept and slope of the growth model of the trait. Since we fit a linear growth model for both DNA methylation and cardiometabolic traits, the factor loading from latent intercept to manifest variable at different time points are all equal to 1, and the factor loading from latent slope to manifest variables are set as 0, 2, 3, 5 and 6 at IPT3, IPT5, IPT6, IPT8 and IPT9, respectively. x1 and x2 are the direct effects from the intercept (representing baseline level) and the slope (representing growth rate) of DNA methylation growth model of one specific CpG to CVD, respectively. x3 and x4 are the direct effects from intercept and slope of one specific cardiometabolic trait growth model to CVD, respectively. x4*m1 represents the indirect path from the baseline level (intercept of the growth model) of DNA methylation to CVD mediated by the trait, x4*m2 represents the indirect path from the growth (slope of growth model) of DNA methylation to CVD mediated by the trait. Variance of mval.i, mval.s, rf.i, rf.s, mval.IPT3-mval.IPT9, rf.IPT3-rf.IPT9 were not displayed in the figure.

## Data Availability

The datasets generated and analyzed during the current study are available in Array Express database of EMBL-EBL (www.ebi.ac.uk/arrayexpress, accession number E-MTAB-7309).

## Abbreviations

ABP1/AOC1: Amine oxidase copper containing 1
ALT-SR: Autoregressive latent trajectory model with structured residuals
BMI: Body mass index
BP: Blood pressure
CpG: 5′-Cytosine-phosphate-guanine-3′
CVD: Cardiovascular disease
DBP: Diastolic blood pressure
DNA: Deoxyribonucleic acid
DZ: Dizygotic twin
EWAS: Epigenome-wide association study
HDL: High-density lipoprotein cholesterol
ICD: International Classification of Diseases
ID: Identity number
IPT: In-person testing
LDL: Low-density lipoprotein cholesterol
meQTL: Methylation quantitative trait loci
MR: Mendelian randomization
MZ: Monozygotic twin
SATSA: Swedish Adoption/Twin Study of Aging
SBP: Systolic blood pressure
SD: Standard deviation
SKI: Ski proto-oncogene
STR: Swedish Twin Registry
TC: Total cholesterol
TG: Total triglyceride
TMUB2: Transmembrane and ubiquitin like domain containing 2
