# Supplementary figures and images for: The epigenetic etiology of cardiovascular disease in a longitudinal Swedish twin study
Source: Clin Epigenetics. 2021 Jun 24;13:129. doi: 10.1186/s13148-021-01113-6 (PMC8223329; doi:10.1186/s13148-021-01113-6)

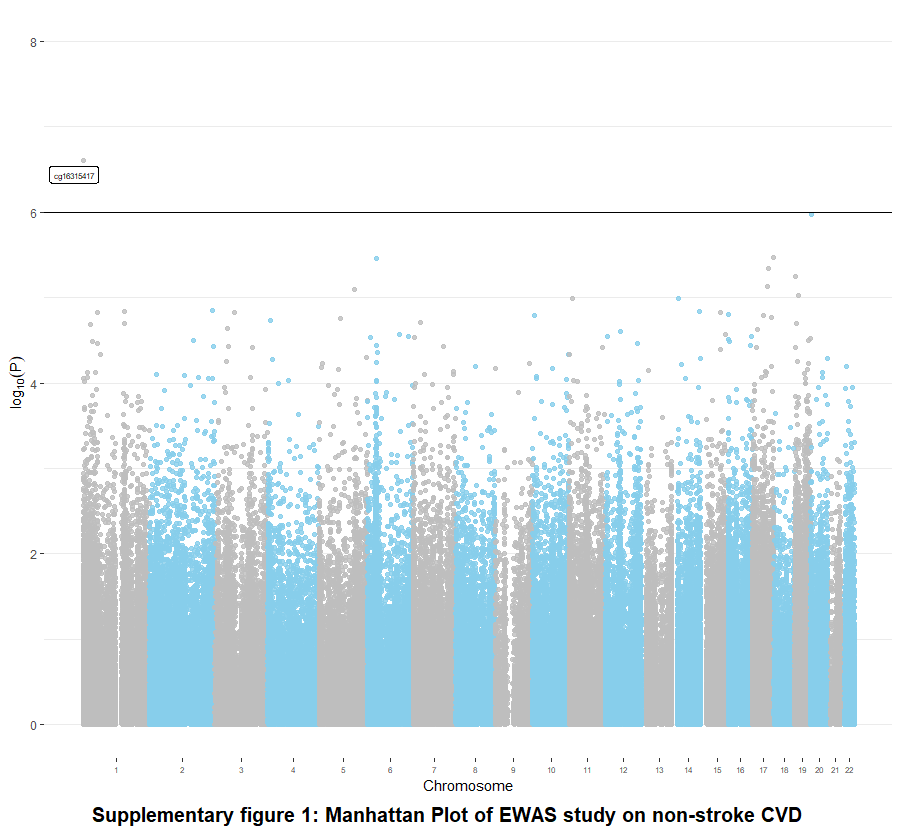

Supplement: Supplementary file 2 — Additional file 2: Figure S1. Manhattan plot of EWAS study on non-stroke CVD. Model was adjusted for age, sex and smoking status. [file 13148_2021_1113_MOESM2_ESM.png]

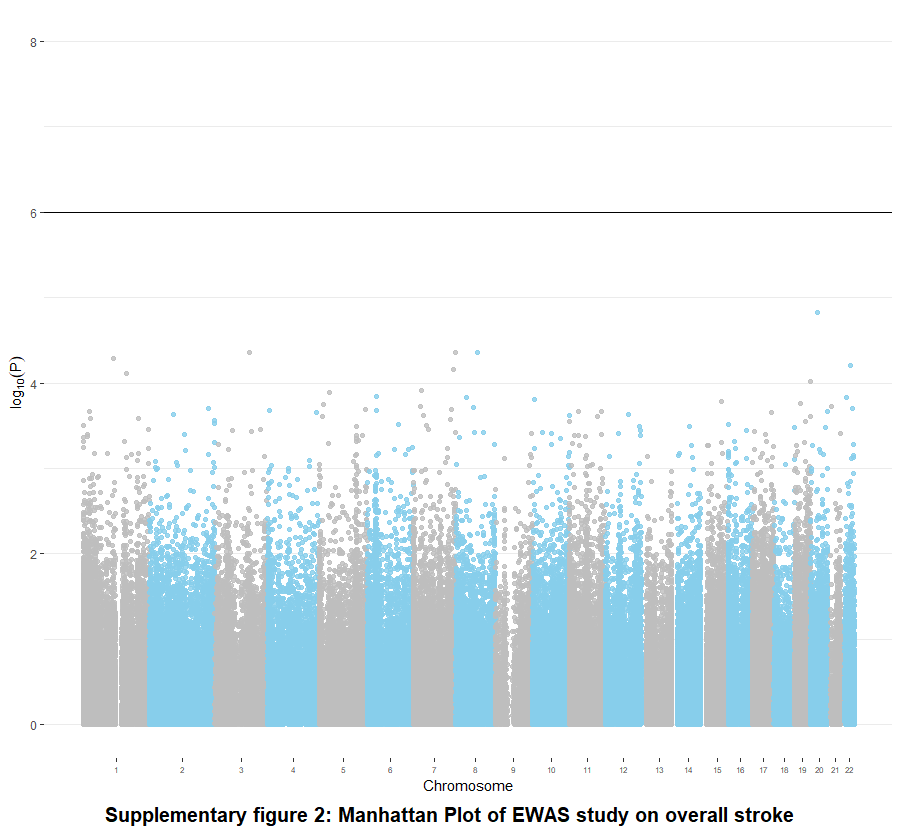

Supplement: Supplementary file 3 — Additional file 3: Figure S2. Manhattan plot of EWAS study on overall stroke. Model was adjusted for age, sex and smoking status. [file 13148_2021_1113_MOESM3_ESM.png]

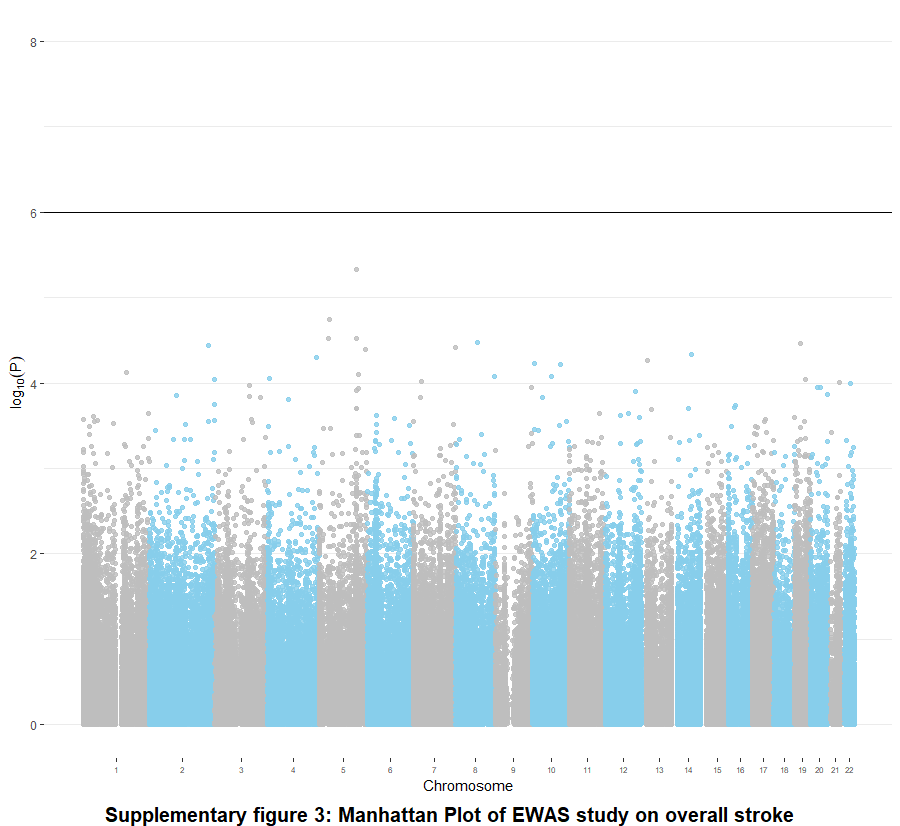

Supplement: Supplementary file 4 — Additional file 4: Figure S3. Manhattan plot of EWAS study on ischemic stroke. Model was adjusted for age, sex and smoking status. [file 13148_2021_1113_MOESM4_ESM.png]

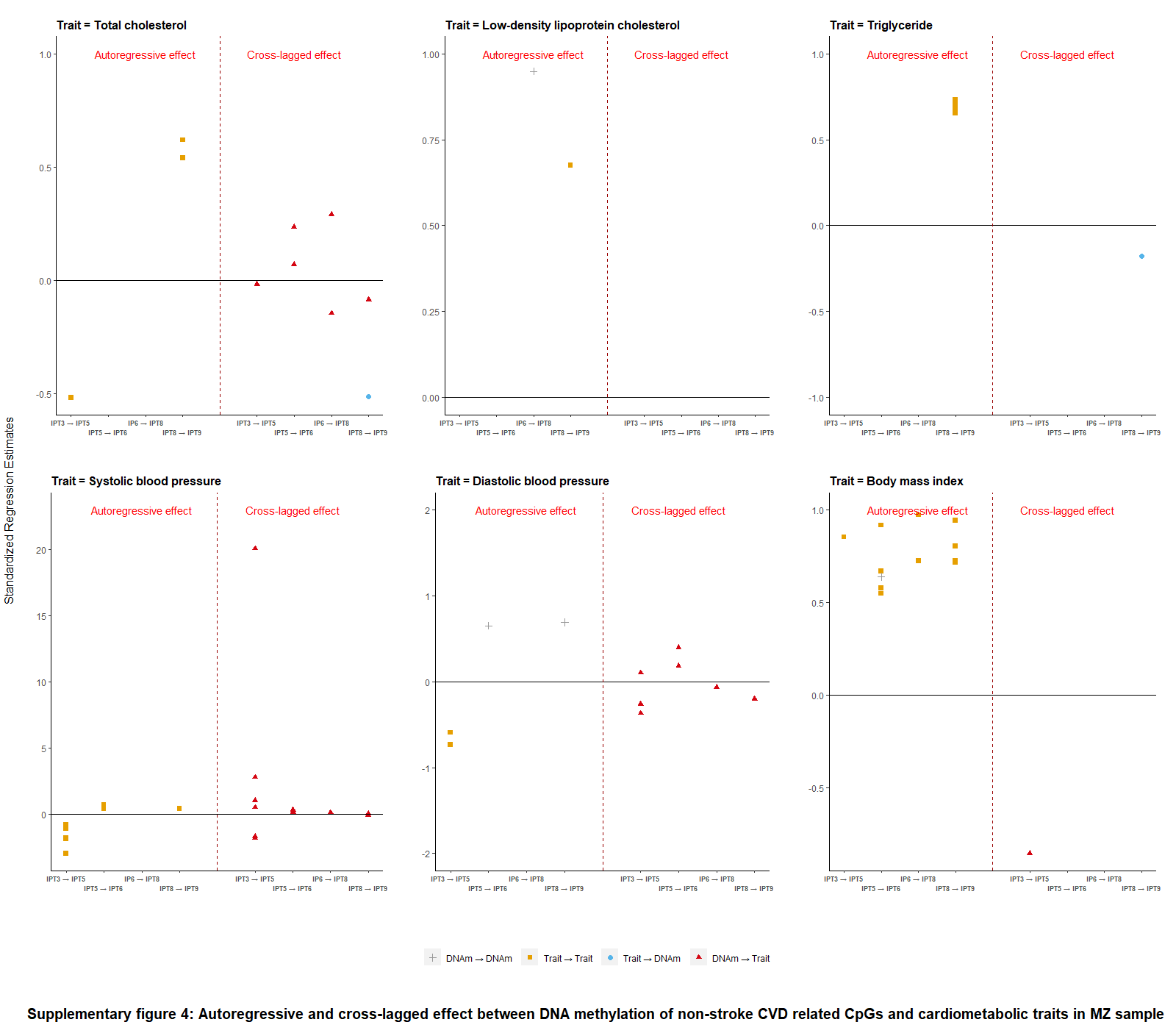

Supplement: Supplementary file 5 — Additional file 5: Figure S4. Autoregressive and cross-lagged effect between DNA methylation of non-stroke CVD-related CpGs and cardiometabolic traits in MZ sample. Each point represents one significant effect (P value was set to 3×10^−4^). The X-axis represents the effect at different adjacent time points, for example, IPT3→IPT5 means the effect of one variable at IPT3 on the other variable at IPT5. The Y-axis represents the standardized estimation coefficient from the regression model. The left part of the figure is the autoregressive effect, and the right part is the cross-lagged effect. “DNAm→DNAm” (gray plus sign) represents autoregressive effect of DNA methylation, “Trait→Trait”(blue circle) represents autoregressive effect of trait, “Trait→DNAm”(brown square) represents cross-lagged effect from trait to DNA methylation, and “DNAm→Trait” (red triangle) represents cross-lagged effect from DNA methylation to trait. MZ, Monozygotic twins; DZ, Dizygotic twins. [file 13148_2021_1113_MOESM5_ESM.png]

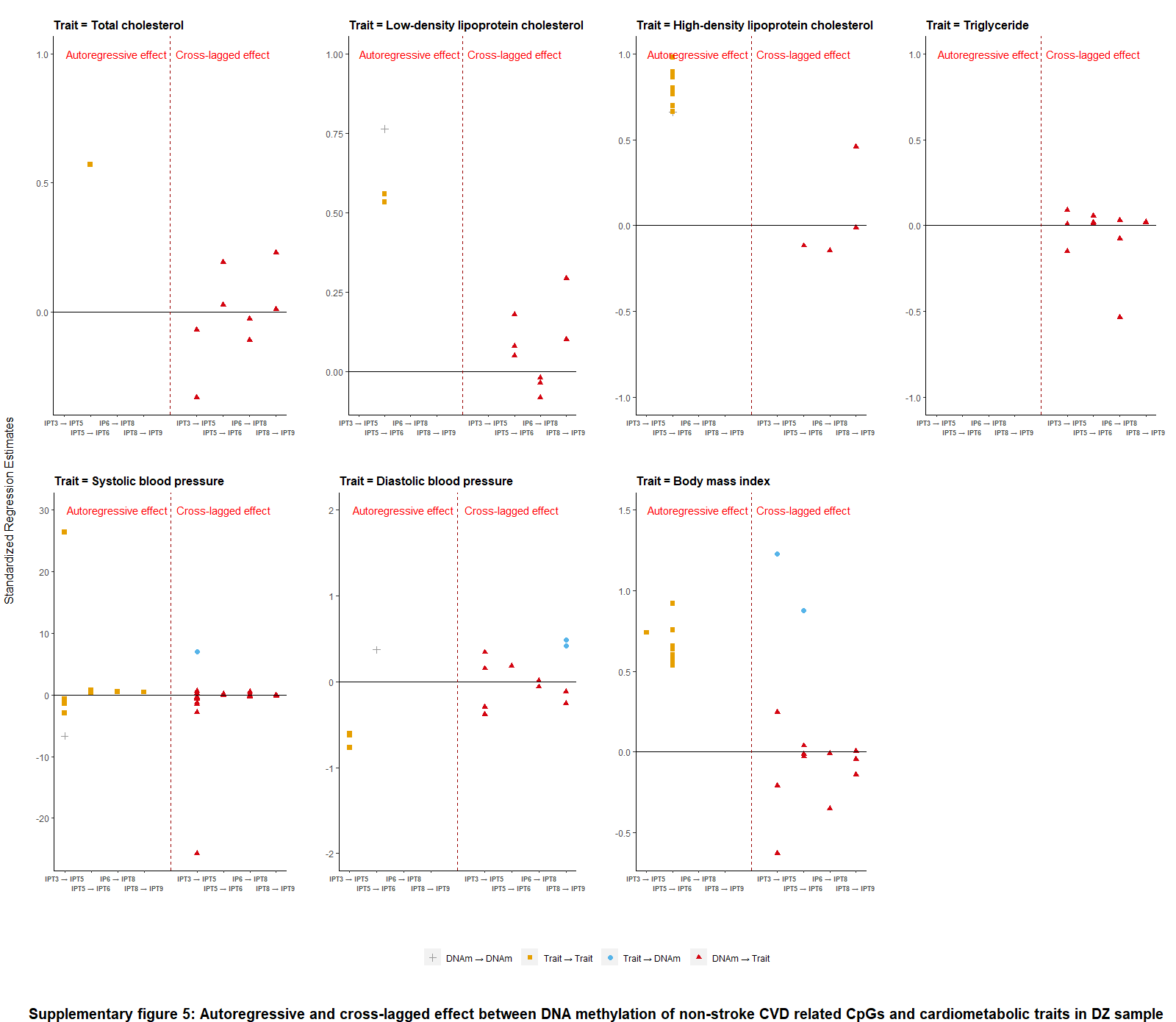

Supplement: Supplementary file 6 — Additional file 6: Figure S5. Autoregressive and cross-lagged effect between DNA methylation of non-stroke CVD-related CpGs and cardiometabolic traits in DZ sample. Each point represents one significant effect (P value was set to 3×10^−4^). The X-axis represents the effect at different adjacent time points, for example, IPT3→IPT5 means the effect of one variable at IPT3 on the other variable at IPT5. The Y-axis represents the standardized estimation coefficient from the regression model. The left part of the figure is the autoregressive effect, and the right part is the cross-lagged effect. “DNAm→DNAm” (gray plus sign) represents autoregressive effect of DNA methylation, “Trait→Trait”(blue circle) represents autoregressive effect of trait, “Trait→DNAm”(brown square) represents cross-lagged effect from trait to DNA methylation, and “DNAm→Trait” (red triangle) represents cross-lagged effect from DNA methylation to trait. MZ, Monozygotic twins; DZ, Dizygotic twins. [file 13148_2021_1113_MOESM6_ESM.png]

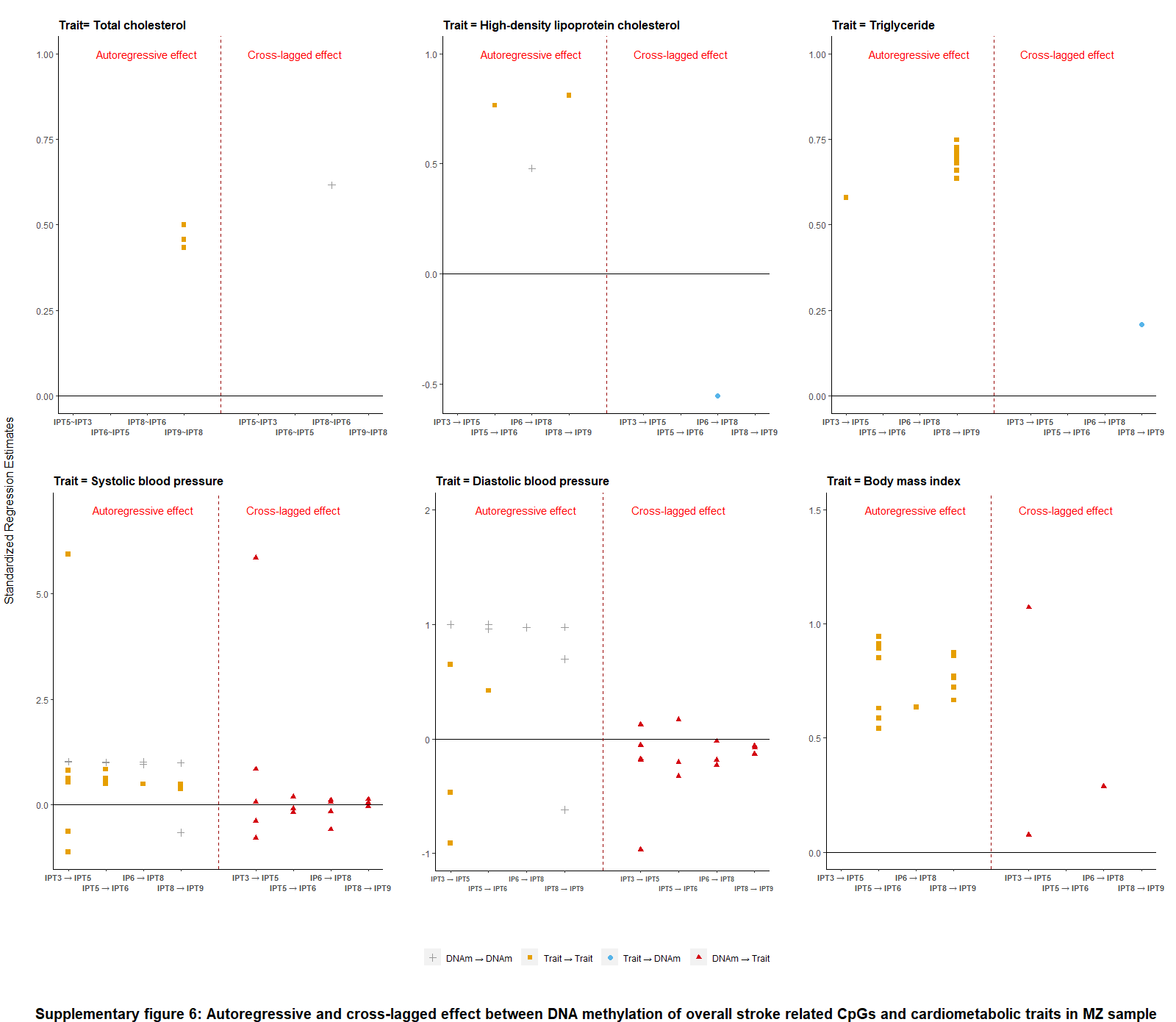

Supplement: Supplementary file 7 — Additional file 7: Figure S6. Autoregressive and cross-lagged effect between DNA methylation of overall stroke-related CpGs and cardiometabolic traits in MZ sample. Each point represents one significant effect (P value was set to 3×10^−4^). The X-axis represents the effect at different adjacent time points, for example, IPT3→IPT5 means the effect of one variable at IPT3 on the other variable at IPT5. The Y-axis represents the standardized estimation coefficient from the regression model. The left part of the figure is the autoregressive effect, and the right part is the cross-lagged effect. “DNAm→DNAm” (gray plus sign) represents autoregressive effect of DNA methylation, “Trait→Trait” (blue circle) represents autoregressive effect of trait, “Trait→DNAm” (brown square) represents cross-lagged effect from trait to DNA methylation, and “DNAm→Trait” (red triangle) represents cross-lagged effect from DNA methylation to trait. MZ, Monozygotic twins; DZ, Dizygotic twins. [file 13148_2021_1113_MOESM7_ESM.png]

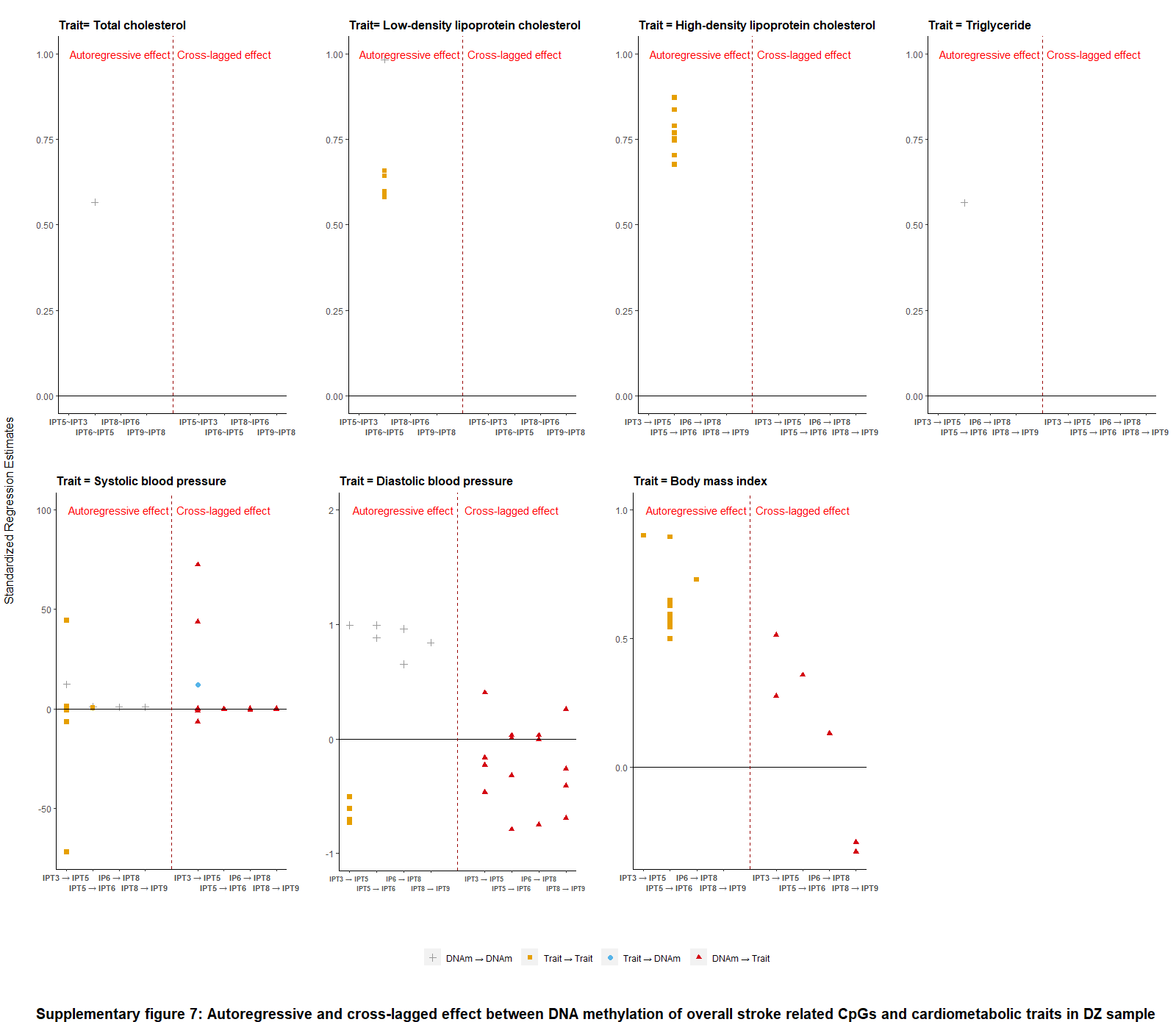

Supplement: Supplementary file 8 — Additional file 8: Figure S7. Autoregressive and cross-lagged effect between DNA methylation of overall stroke-related CpGs and cardiometabolic traits in DZ sample. Each point represents one significant effect (P value was set to 3×10^−4^). The X-axis represents the effect at different adjacent time points, for example, IPT3→IPT5 means the effect of one variable at IPT3 on the other variable at IPT5. The Y-axis represents the standardized estimation coefficient from the regression model. The left part of the figure is the autoregressive effect, and the right part is the cross-lagged effect. “DNAm→DNAm” (gray plus sign) represents autoregressive effect of DNA methylation, “Trait→Trait” (blue circle) represents autoregressive effect of trait, “Trait→DNAm” (brown square) represents cross-lagged effect from trait to DNA methylation, and “DNAm→Trait” (red triangle) represents cross-lagged effect from DNA methylation to trait. MZ, Monozygotic twins; DZ, Dizygotic twins. [file 13148_2021_1113_MOESM8_ESM.png]

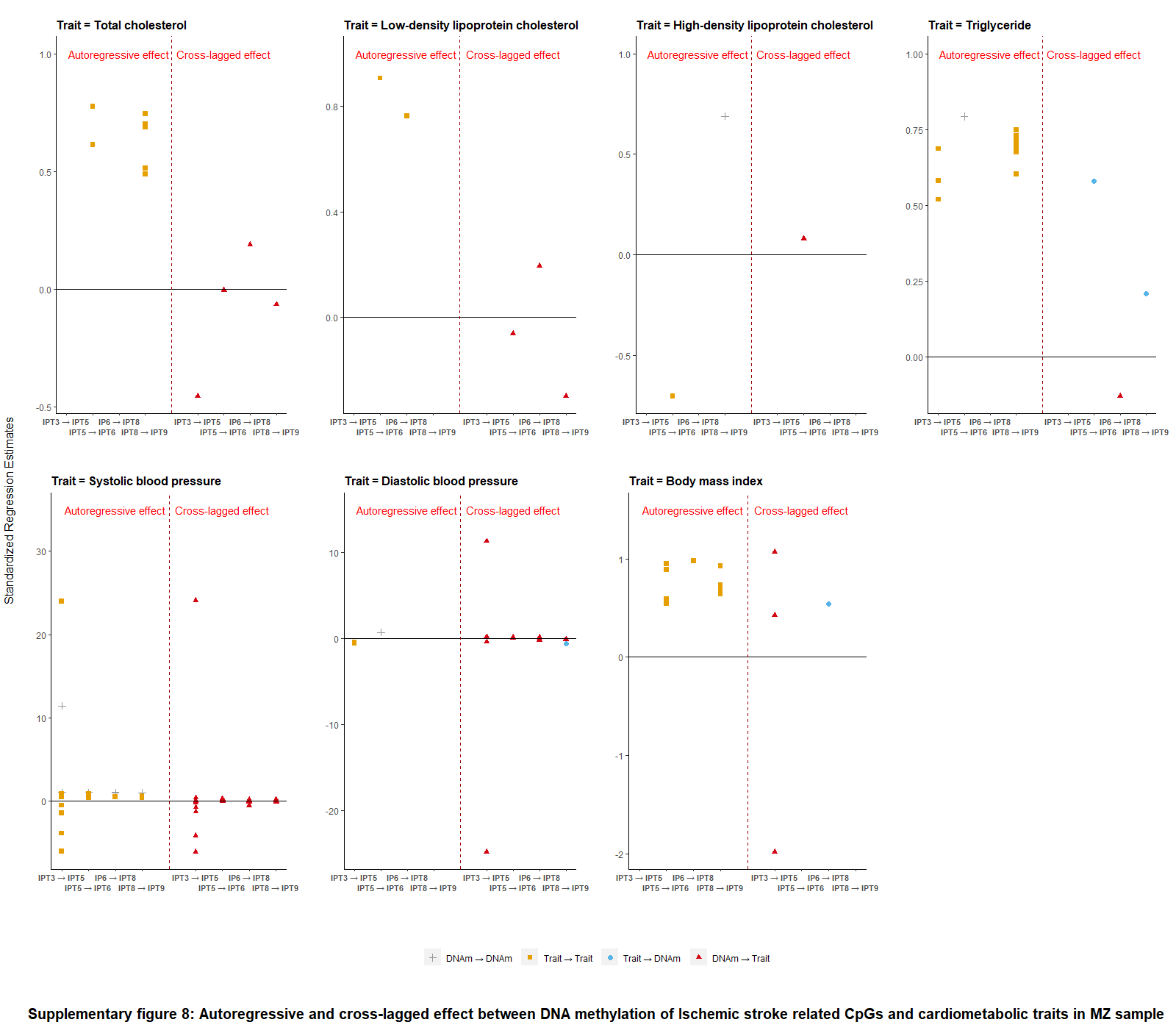

Supplement: Supplementary file 9 — Additional file 9: Figure S8. Autoregressive and cross-lagged effect between DNA methylation of ischemic stroke-related CpGs and cardiometabolic traits in MZ sample. Each point represents one significant effect (P value was set to 3×10^−4^). The X-axis represents the effect at different adjacent time points, for example, IPT3→IPT5 means the effect of one variable at IPT3 on the other variable at IPT5. The Y-axis represents the standardized estimation coefficient from the regression model. The left part of the figure is the autoregressive effect, and the right part is the cross-lagged effect. “DNAm→DNAm” (gray plus sign) represents autoregressive effect of DNA methylation, “Trait→Trait” (blue circle) represents autoregressive effect of trait, “Trait→DNAm” (brown square) represents cross-lagged effect from trait to DNA methylation, and “DNAm→Trait” (red triangle) represents cross-lagged effect from DNA methylation to trait. MZ, Monozygotic twins; DZ, Dizygotic twins. [file 13148_2021_1113_MOESM9_ESM.png]

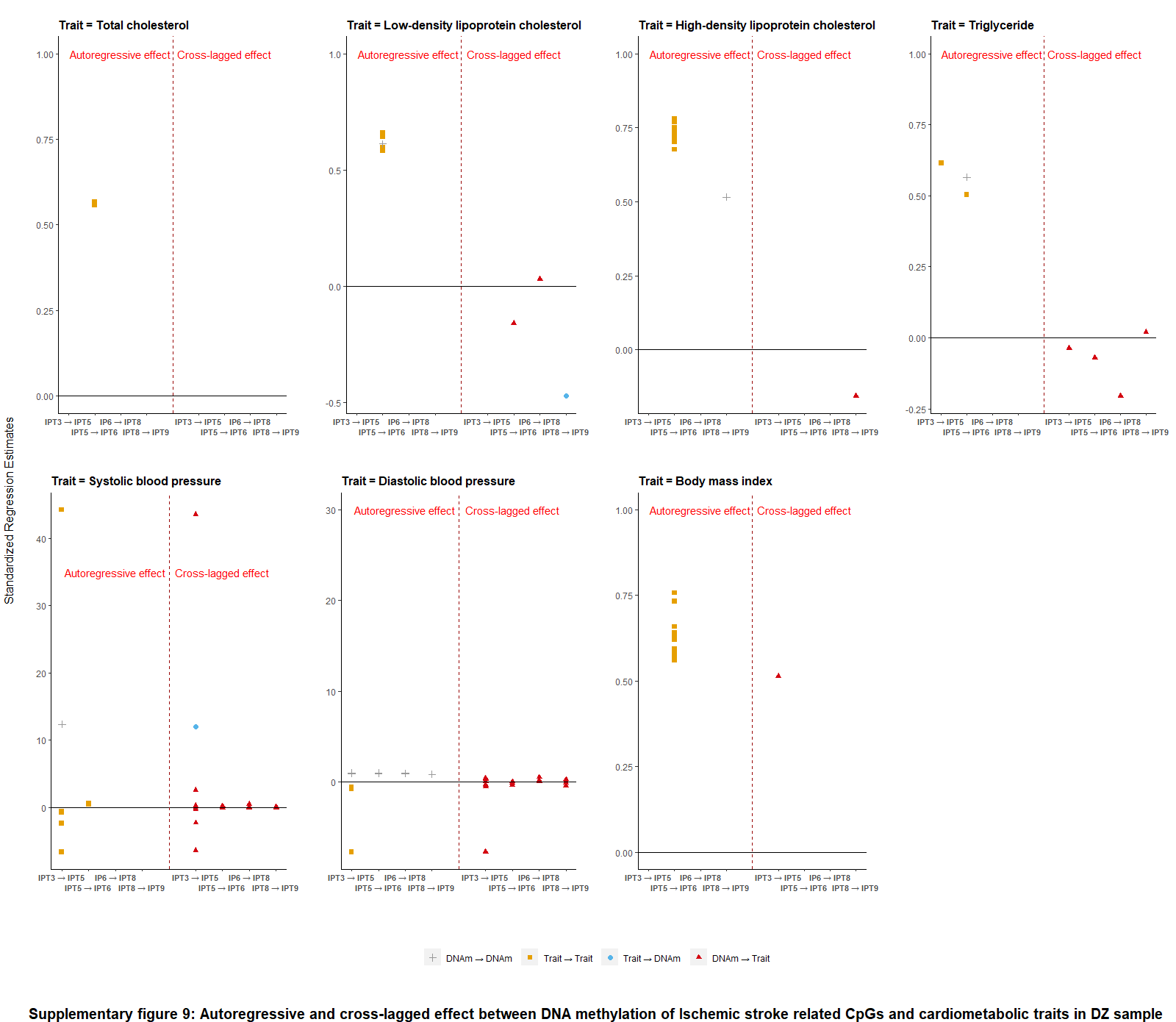

Supplement: Supplementary file 10 — Additional file 10: Figure S9. Autoregressive and cross-lagged effect between DNA methylation of ischemic stroke-related CpGs and cardiometabolic traits in DZ sample. Each point represents one significant effect (P value was set to 3×10^−4^). The X-axis represents the effect at different adjacent time points, for example, IPT3→IPT5 means the effect of one variable at IPT3 on the other variable at IPT5. The Y-axis represents the standardized estimation coefficient from the regression model. The left part of the figure is the autoregressive effect, and the right part is the cross-lagged effect. “DNAm→DNAm” (gray plus sign) represents autoregressive effect of DNA methylation, “Trait→Trait” (blue circle) represents autoregressive effect of trait, “Trait→DNAm” (brown square) represents cross-lagged effect from trait to DNA methylation, and “DNAm→Trait” (red triangle) represents cross-lagged effect from DNA methylation to trait. MZ, Monozygotic twins; DZ, Dizygotic twins. [file 13148_2021_1113_MOESM10_ESM.png]

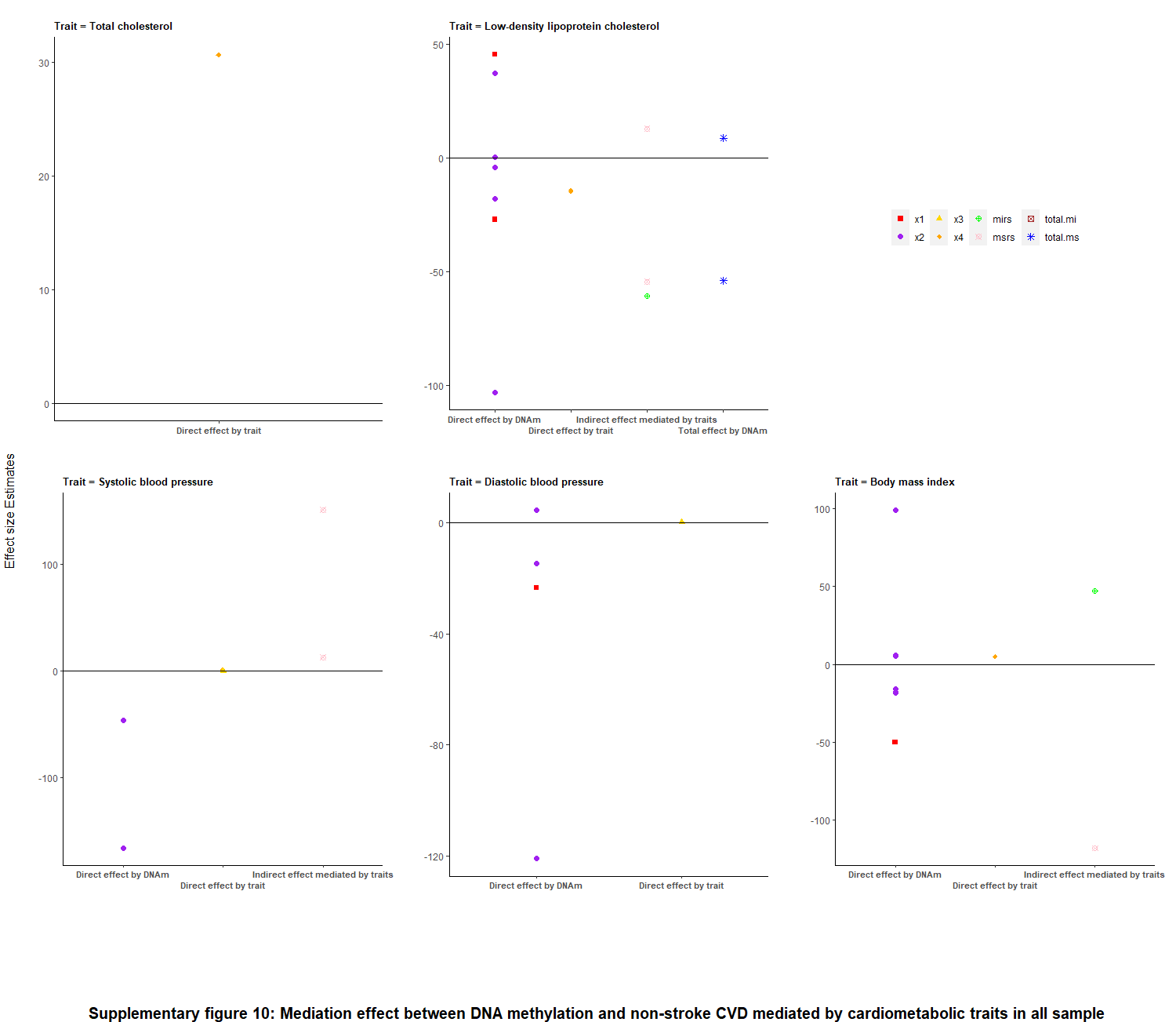

Supplement: Supplementary file 11 — Additional file 11: Figure S10. Mediation effect between DNA methylation and non-stroke CVD mediated by cardiometabolic traits in all sample. Each point represents one significant effect (P value was set to 3×10^−4^ for multiple testing) identified from mediation analysis among one specific CpG site, one specific cardiometabolic trait, and one specific outcome. The x axis represents the categories of direct effect, indirect effect and total effect, and the y axis represents the estimates of the three effects. "x1” (red) and “x2” (purple) represent direct effect from the intercept and the slope of DNA methylation at one specific CpG to CVD, respectively. “x3” (gold) and “x4” (orange) represent the direct effect from the intercept and the slope of one specific trait to CVD, respectively. “mirs” (x4*m1 in the mediation model, green) represents the indirect effect from the intercept of DNA methylation at one specific CpG to CVD mediated by one specific trait. “msrs” (x4*m2 in the mediation model, pink) represents the indirect effect from the slope of DNA methylation at one specific CpG to CVD mediated by one specific trait. “total_mi” (brown) represents the total effect from the intercept of DNA methylation at one specific CpG to CVD and equals to “x1+x4*m1”. “total_ms” (blue) represents the total effect from the slope of DNA methylation at one specific CpG to CVD and equals to “x2+x4*m2”. [file 13148_2021_1113_MOESM11_ESM.png]

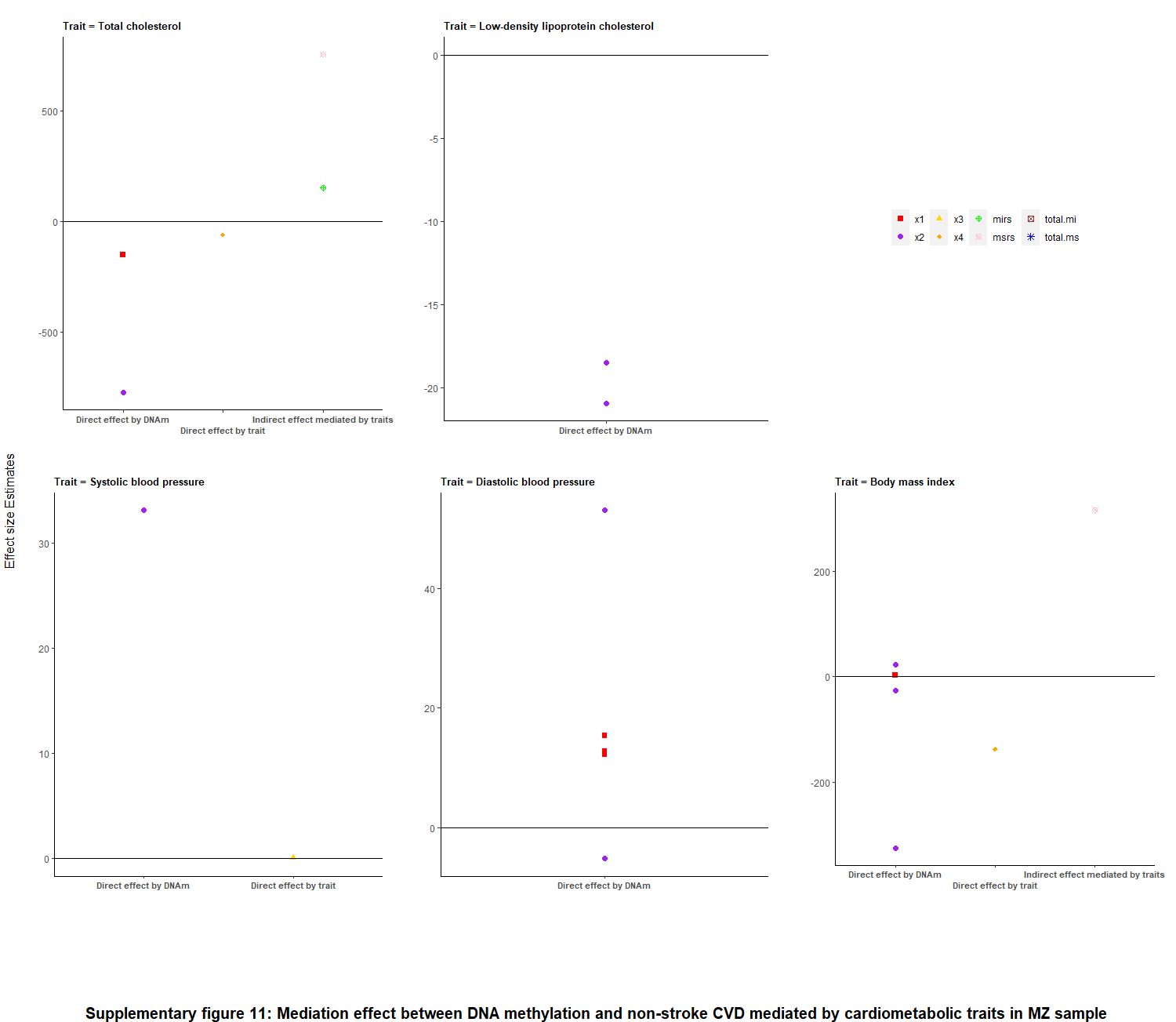

Supplement: Supplementary file 12 — Additional file 12: Figure S11. Mediation effect between DNA methylation and non-stroke CVD mediated by cardiometabolic traits in MZ sample. Each point represents one significant effect (P value was set to 3×10^−4^ for multiple testing) identified from mediation analysis among one specific CpG site, one specific cardiometabolic trait, and one specific outcome. The x axis represents the categories of direct effect, indirect effect and total effect, and the y axis represents the estimates of the three effects. "x1” (red) and “x2” (purple) represent direct effect from the intercept and the slope of DNA methylation at one specific CpG to CVD, respectively. “x3” (gold) and “x4” (orange) represent the direct effect from the intercept and the slope of one specific trait to CVD, respectively. “mirs” (x4*m1 in the mediation model, green) represents the indirect effect from the intercept of DNA methylation at one specific CpG to CVD mediated by one specific trait. “msrs” (x4*m2 in the mediation model, pink) represents the indirect effect from the slope of DNA methylation at one specific CpG to CVD mediated by one specific trait. “total_mi” (brown) represents the total effect from the intercept of DNA methylation at one specific CpG to CVD and equals to “x1+x4*m1”. “total_ms” (blue) represents the total effect from the slope of DNA methylation at one specific CpG to CVD and equals to “x2+x4*m2”. MZ, Monozygotic twins; DZ, Dizygotic twins. [file 13148_2021_1113_MOESM12_ESM.png]

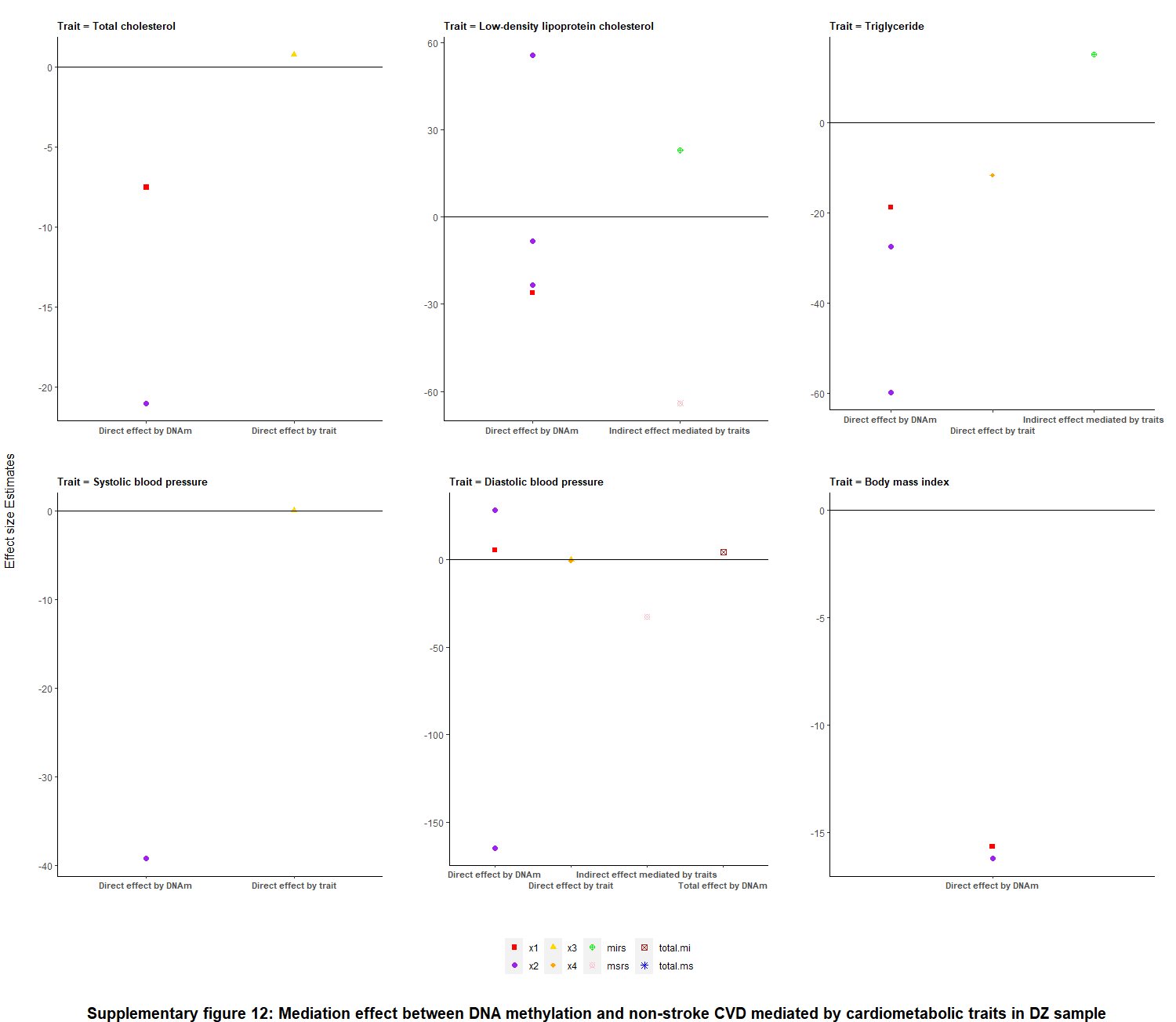

Supplement: Supplementary file 13 — Additional file 13: Figure S12. Mediation effect between DNA methylation and non-stroke CVD mediated by cardiometabolic traits in DZ sample. Each point represents one significant effect (P value was set to 3×10^−4^ for multiple testing) identified from mediation analysis among one specific CpG site, one specific cardiometabolic trait, and one specific outcome. The x axis represents the categories of direct effect, indirect effect and total effect, and the y axis represents the estimates of the three effects. "x1” (red) and “x2” (purple) represent direct effect from the intercept and the slope of DNA methylation at one specific CpG to CVD, respectively. “x3” (gold) and “x4” (orange) represent the direct effect from the intercept and the slope of one specific trait to CVD, respectively. “mirs” (x4*m1 in the mediation model, green) represents the indirect effect from the intercept of DNA methylation at one specific CpG to CVD mediated by one specific trait. “msrs” (x4*m2 in the mediation model, pink) represents the indirect effect from the slope of DNA methylation at one specific CpG to CVD mediated by one specific trait. “total_mi” (brown) represents the total effect from the intercept of DNA methylation at one specific CpG to CVD and equals to “x1+x4*m1”. “total_ms” (blue) represents the total effect from the slope of DNA methylation at one specific CpG to CVD and equals to “x2+x4*m2”. MZ, Monozygotic twins; DZ, Dizygotic twins. [file 13148_2021_1113_MOESM13_ESM.png]

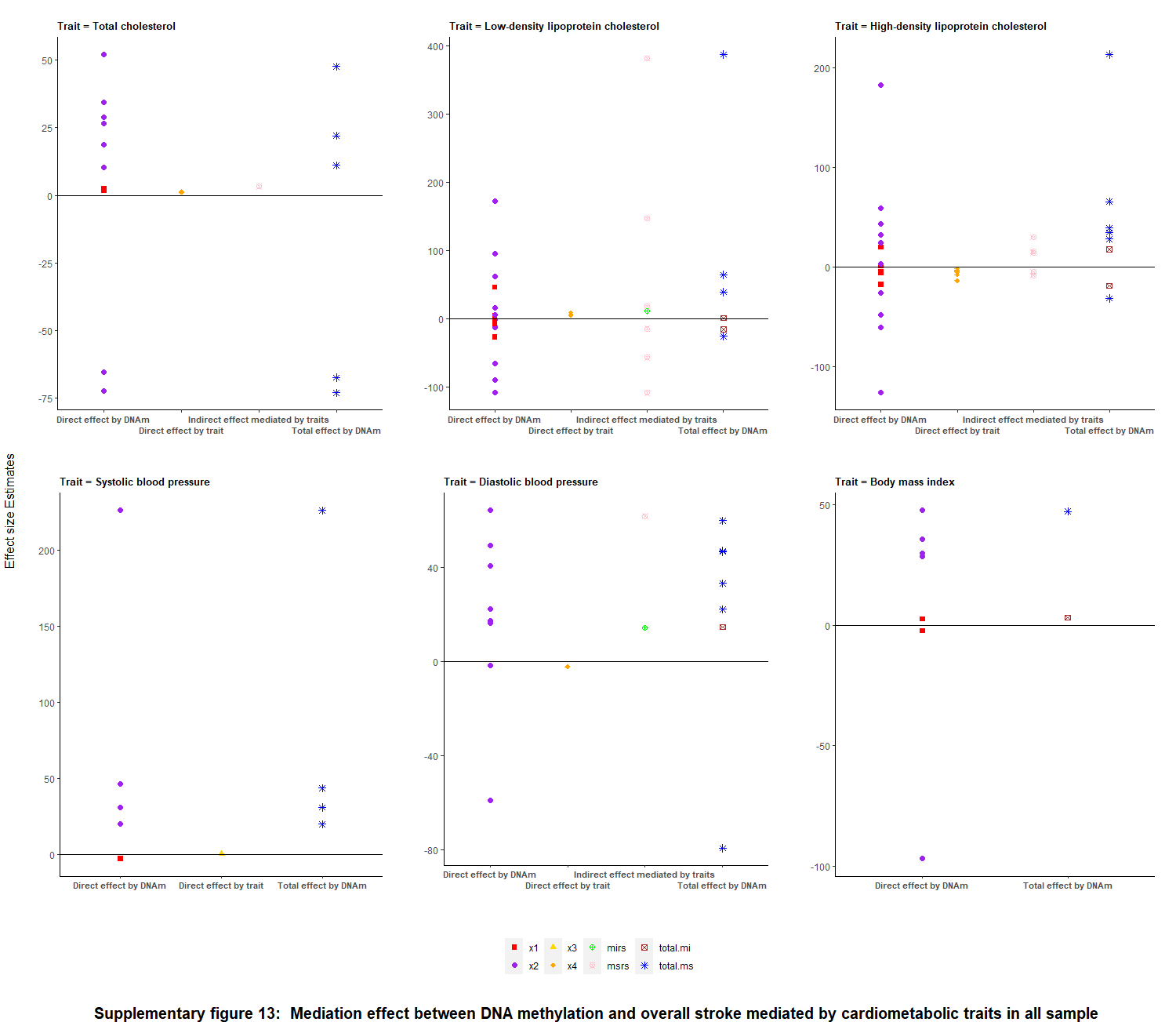

Supplement: Supplementary file 14 — Additional file 14: Figure S13. Mediation effect between DNA methylation and overall stroke mediated by cardiometabolic traits in all sample. Each point represents one significant effect (P value was set to 3×10^−4^ for multiple testing) identified from mediation analysis among one specific CpG site, one specific cardiometabolic trait, and one specific outcome. The x axis represents the categories of direct effect, indirect effect and total effect, and the y axis represents the estimates of the three effects. "x1” (red) and “x2” (purple) represent direct effect from the intercept and the slope of DNA methylation at one specific CpG to CVD, respectively. “x3” (gold) and “x4” (orange) represent the direct effect from the intercept and the slope of one specific trait to CVD, respectively. “mirs” (x4*m1 in the mediation model, green) represents the indirect effect from the intercept of DNA methylation at one specific CpG to CVD mediated by one specific trait. “msrs” (x4*m2 in the mediation model, pink) represents the indirect effect from the slope of DNA methylation at one specific CpG to CVD mediated by one specific trait. “total_mi” (brown) represents the total effect from the intercept of DNA methylation at one specific CpG to CVD and equals to “x1+x4*m1”. “total_ms” (blue) represents the total effect from the slope of DNA methylation at one specific CpG to CVD and equals to “x2+x4*m2”. [file 13148_2021_1113_MOESM14_ESM.png]

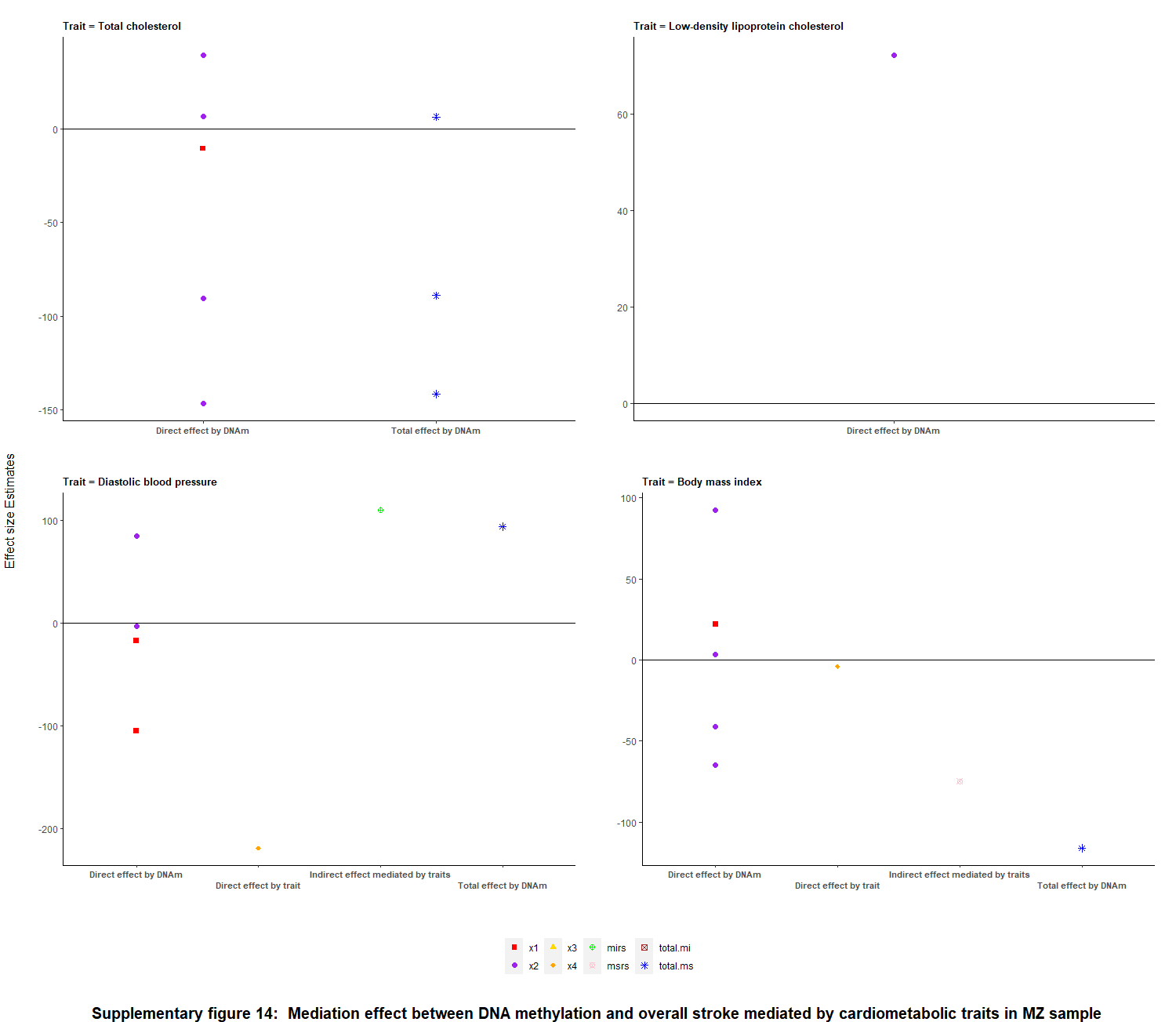

Supplement: Supplementary file 15 — Additional file 15: Figure S14. Mediation effect between DNA methylation and overall stroke mediated by cardiometabolic traits in MZ sample. Each point represents one significant effect (P value was set to 3×10^−4^ for multiple testing) identified from mediation analysis among one specific CpG site, one specific cardiometabolic trait, and one specific outcome. The x axis represents the categories of direct effect, indirect effect and total effect, and the y axis represents the estimates of the three effects. "x1” (red) and “x2” (purple) represent direct effect from the intercept and the slope of DNA methylation at one specific CpG to CVD, respectively. “x3” (gold) and “x4” (orange) represent the direct effect from the intercept and the slope of one specific trait to CVD, respectively. “mirs” (x4*m1 in the mediation model, green) represents the indirect effect from the intercept of DNA methylation at one specific CpG to CVD mediated by one specific trait. “msrs” (x4*m2 in the mediation model, pink) represents the indirect effect from the slope of DNA methylation at one specific CpG to CVD mediated by one specific trait. “total_mi” (brown) represents the total effect from the intercept of DNA methylation at one specific CpG to CVD and equals to “x1+x4*m1”. “total_ms” (blue) represents the total effect from the slope of DNA methylation at one specific CpG to CVD and equals to “x2+x4*m2”. MZ, Monozygotic twins; DZ, Dizygotic twins. [file 13148_2021_1113_MOESM15_ESM.png]

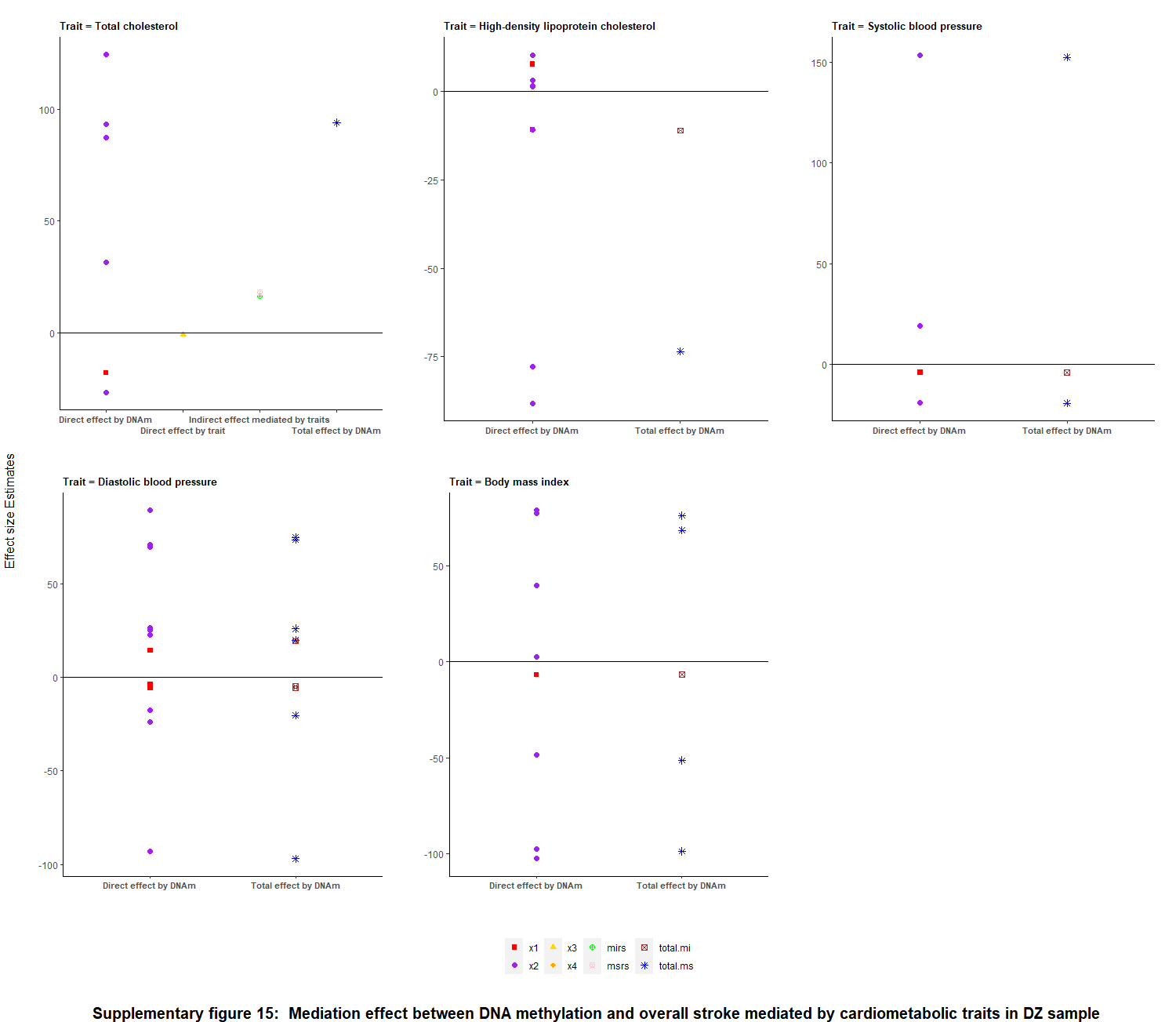

Supplement: Supplementary file 16 — Additional file 16: Figure S15. Mediation effect between DNA methylation and overall stroke mediated by cardiometabolic traits in DZ sample. Each point represents one significant effect (P value was set to 3×10^−4^ for multiple testing) identified from mediation analysis among one specific CpG site, one specific cardiometabolic trait, and one specific outcome. The x axis represents the categories of direct effect, indirect effect and total effect, and the y axis represents the estimates of the three effects. "x1” (red) and “x2” (purple) represent direct effect from the intercept and the slope of DNA methylation at one specific CpG to CVD, respectively. “x3” (gold) and “x4” (orange) represent the direct effect from the intercept and the slope of one specific trait to CVD, respectively. “mirs” (x4*m1 in the mediation model, green) represents the indirect effect from the intercept of DNA methylation at one specific CpG to CVD mediated by one specific trait. “msrs” (x4*m2 in the mediation model, pink) represents the indirect effect from the slope of DNA methylation at one specific CpG to CVD mediated by one specific trait. “total_mi” (brown) represents the total effect from the intercept of DNA methylation at one specific CpG to CVD and equals to “x1+x4*m1”. “total_ms” (blue) represents the total effect from the slope of DNA methylation at one specific CpG to CVD and equals to “x2+x4*m2”. MZ, Monozygotic twins; DZ, Dizygotic twins. [file 13148_2021_1113_MOESM16_ESM.png]

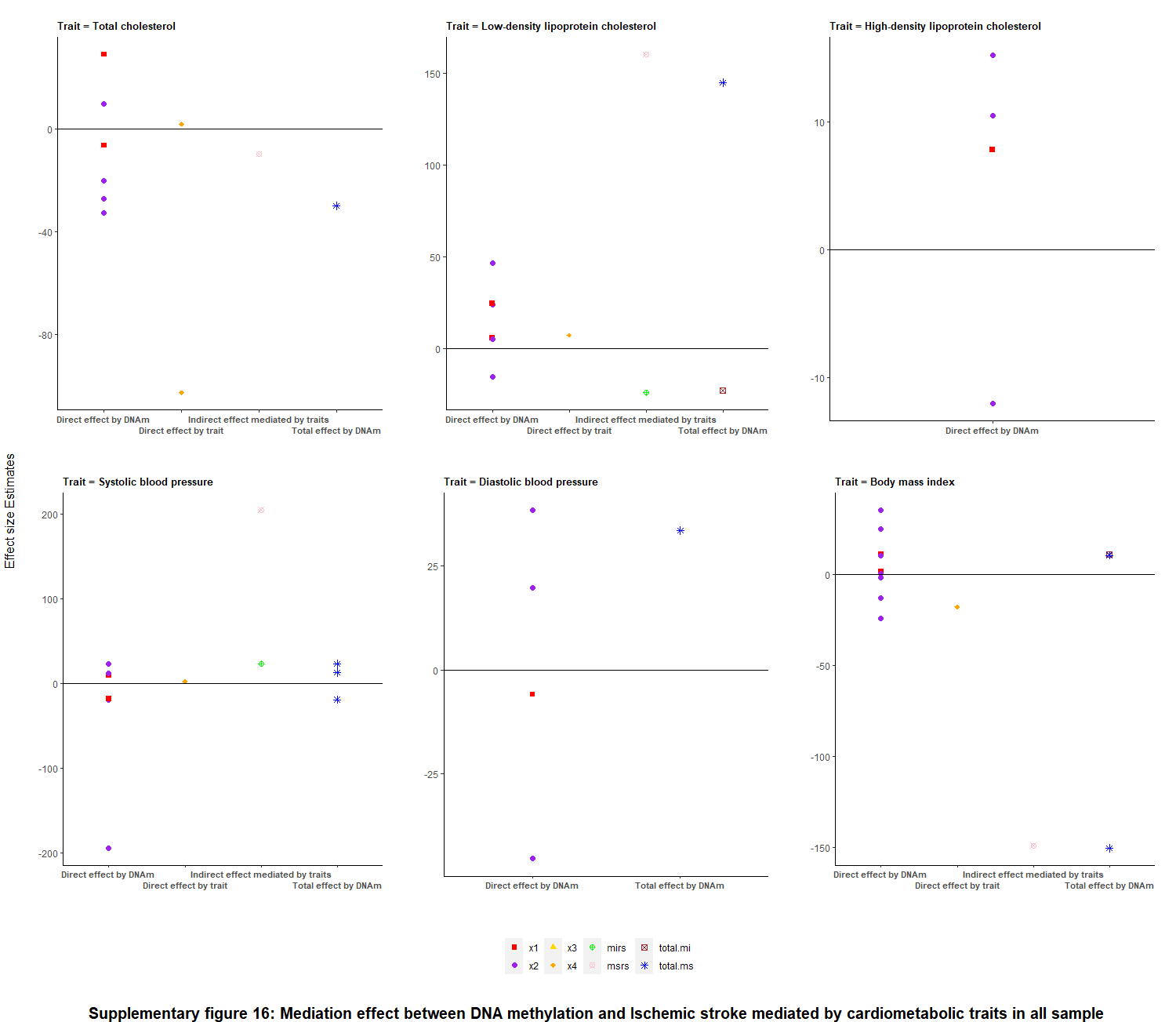

Supplement: Supplementary file 17 — Additional file 17: Figure S16. Mediation effect between DNA methylation and ischemic stroke mediated by cardiometabolic traits in all sample. Each point represents one significant effect (P value was set to 3×10^−4^ for multiple testing) identified from mediation analysis among one specific CpG site, one specific cardiometabolic trait, and one specific outcome. The x axis represents the categories of direct effect, indirect effect and total effect, and the y axis represents the estimates of the three effects. "x1” (red) and “x2” (purple) represent direct effect from the intercept and the slope of DNA methylation at one specific CpG to CVD, respectively. “x3” (gold) and “x4” (orange) represent the direct effect from the intercept and the slope of one specific trait to CVD, respectively. “mirs” (x4*m1 in the mediation model, green) represents the indirect effect from the intercept of DNA methylation at one specific CpG to CVD mediated by one specific trait. “msrs” (x4*m2 in the mediation model, pink) represents the indirect effect from the slope of DNA methylation at one specific CpG to CVD mediated by one specific trait. “total_mi” (brown) represents the total effect from the intercept of DNA methylation at one specific CpG to CVD and equals to “x1+x4*m1”. “total_ms” (blue) represents the total effect from the slope of DNA methylation at one specific CpG to CVD and equals to “x2+x4*m2”. [file 13148_2021_1113_MOESM17_ESM.png]

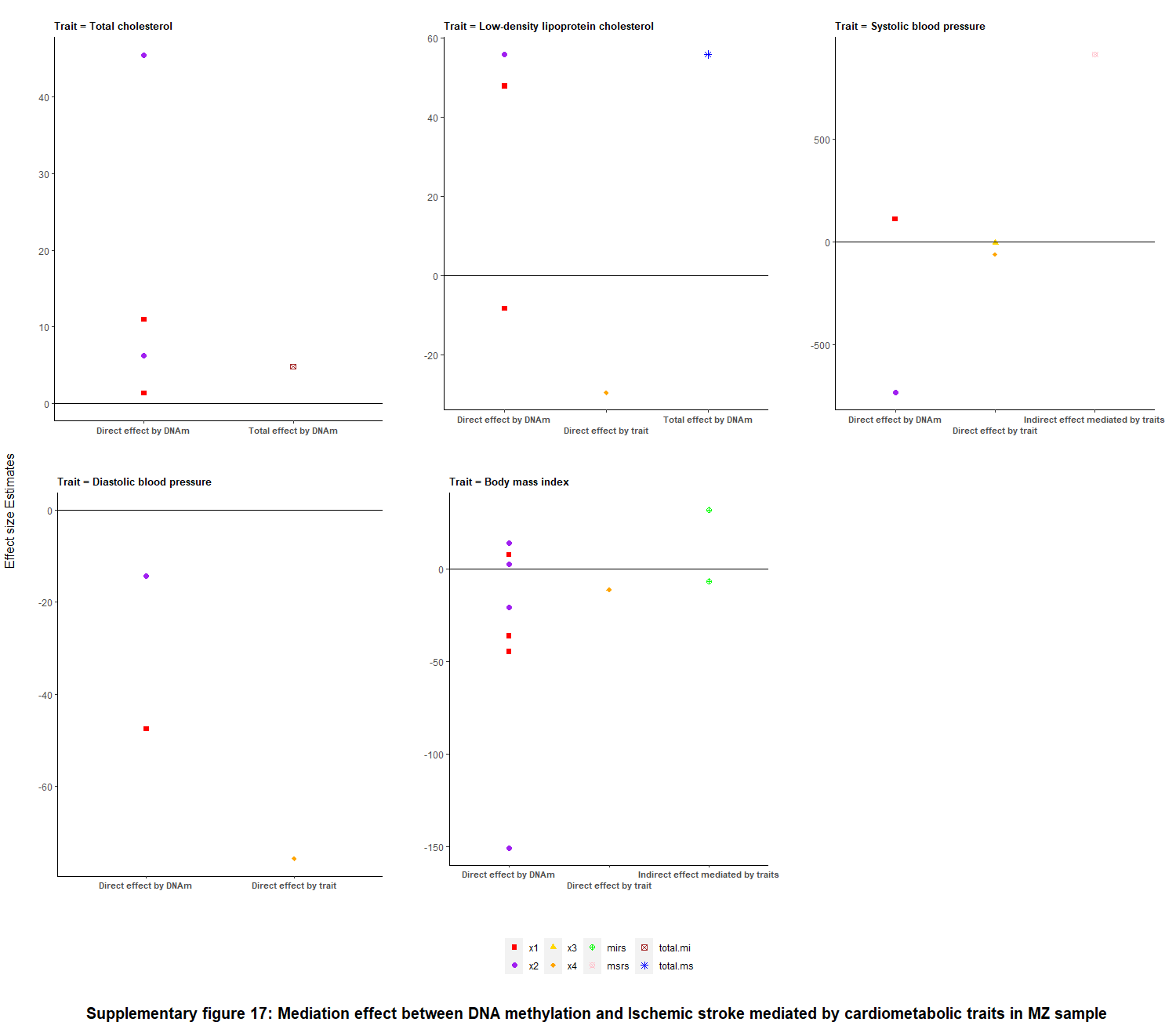

Supplement: Supplementary file 18 — Additional file 18: Figure S17. Mediation effect between DNA methylation and ischemic stroke mediated by cardiometabolic traits in MZ sample. Each point represents one significant effect (P value was set to 3×10^−4^ for multiple testing) identified from mediation analysis among one specific CpG site, one specific cardiometabolic trait, and one specific outcome. The x axis represents the categories of direct effect, indirect effect and total effect, and the y axis represents the estimates of the three effects. "x1” (red) and “x2” (purple) represent direct effect from the intercept and the slope of DNA methylation at one specific CpG to CVD, respectively. “x3” (gold) and “x4” (orange) represent the direct effect from the intercept and the slope of one specific trait to CVD, respectively. “mirs” (x4*m1 in the mediation model, green) represents the indirect effect from the intercept of DNA methylation at one specific CpG to CVD mediated by one specific trait. “msrs” (x4*m2 in the mediation model, pink) represents the indirect effect from the slope of DNA methylation at one specific CpG to CVD mediated by one specific trait. “total_mi” (brown) represents the total effect from the intercept of DNA methylation at one specific CpG to CVD and equals to “x1+x4*m1”. “total_ms” (blue) represents the total effect from the slope of DNA methylation at one specific CpG to CVD and equals to “x2+x4*m2”. MZ, Monozygotic twins; DZ, Dizygotic twins. [file 13148_2021_1113_MOESM18_ESM.png]

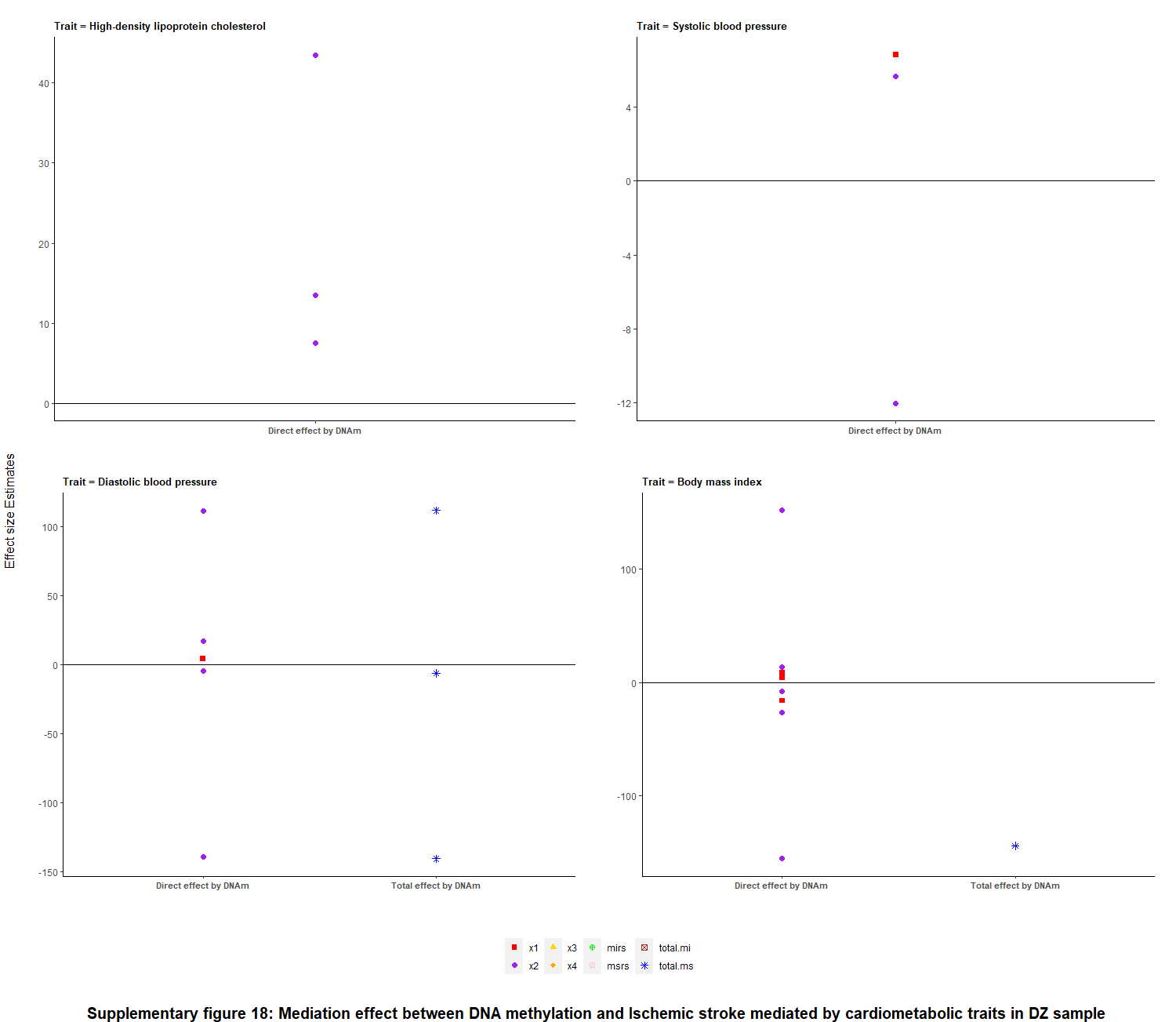

Supplement: Supplementary file 19 — Additional file 19: Figure S18. Mediation effect between DNA methylation and ischemic stroke mediated by cardiometabolic traits in DZ sample. Each point represents one significant effect (P value was set to 3×10^−4^ for multiple testing) identified from mediation analysis among one specific CpG site, one specific cardiometabolic trait, and one specific outcome. The x axis represents the categories of direct effect, indirect effect and total effect, and the y axis represents the estimates of the three effects. "x1” (red) and “x2” (purple) represent direct effect from the intercept and the slope of DNA methylation at one specific CpG to CVD, respectively. “x3” (gold) and “x4” (orange) represent the direct effect from the intercept and the slope of one specific trait to CVD, respectively. “mirs” (x4*m1 in the mediation model, green) represents the indirect effect from the intercept of DNA methylation at one specific CpG to CVD mediated by one specific trait. “msrs” (x4*m2 in the mediation model, pink) represents the indirect effect from the slope of DNA methylation at one specific CpG to CVD mediated by one specific trait. “total_mi” (brown) represents the total effect from the intercept of DNA methylation at one specific CpG to CVD and equals to “x1+x4*m1”. “total_ms” (blue) represents the total effect from the slope of DNA methylation at one specific CpG to CVD and equals to “x2+x4*m2”. MZ, Monozygotic twins; DZ, Dizygotic twins. [file 13148_2021_1113_MOESM19_ESM.png]

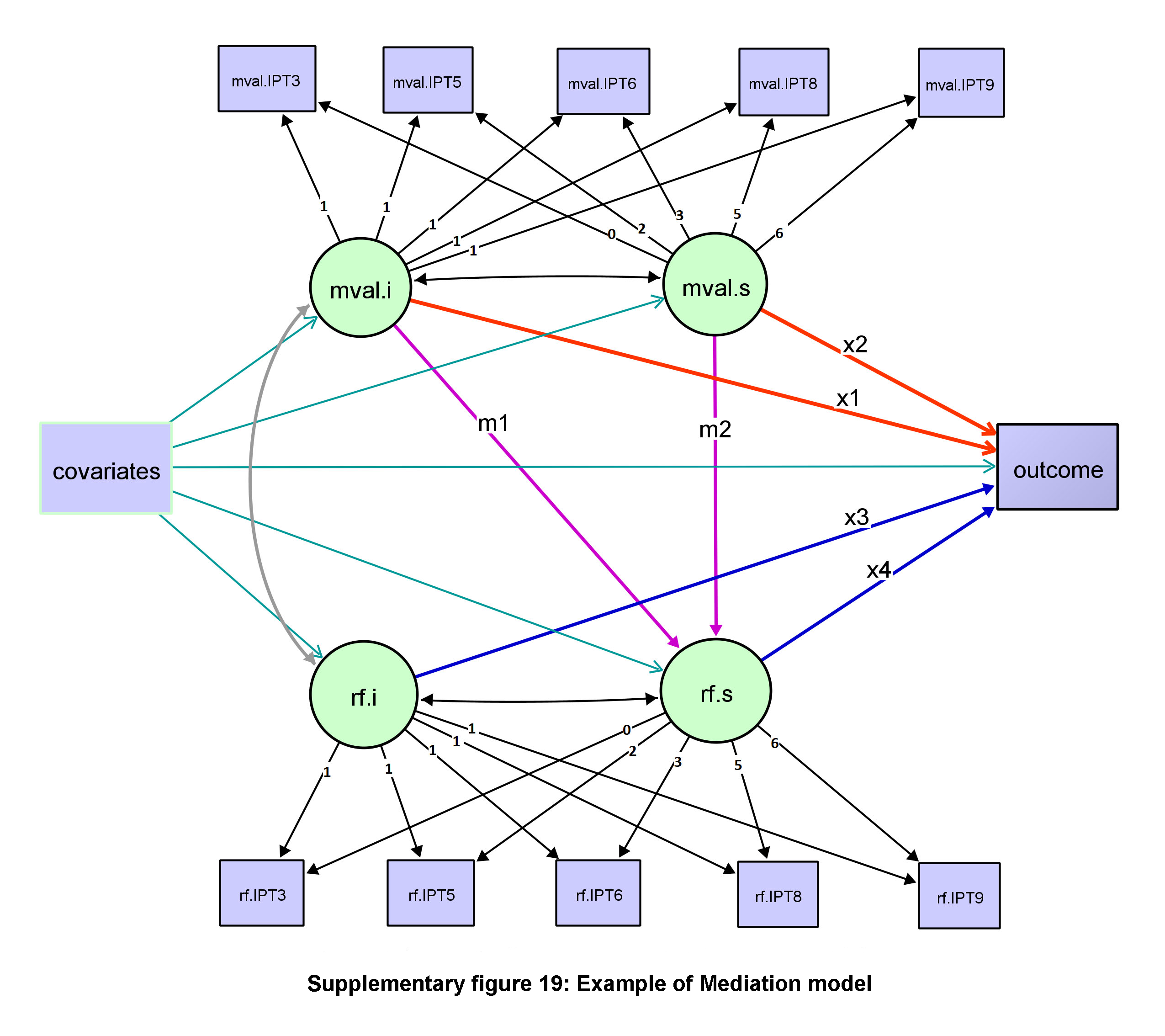

Supplement: Supplementary file 20 — Additional file 20: Figure S19. Example of Mediation model. This figure demonstrates an example of the mediation path assuming that the effect of DNA methylation on CVD (outcome) is mediated by one specific cardiometabolic trait. A mediation path is established in the framework of LGM, that is DNA methylation and trait grow by time, the random intercept of the two constructs correlate with each other, the mediation path is established connecting DNA methylation (either intercept or slope of DNA methylation growth curve) to CVD with the slope of cardiometabolic traits as mediators. Besides of the mediation path indicating the indirect effect of one construct on the outcome mediated by the other construct, the direct effects path is established from the parameters of LGM of either construct to outcome. We include sex and baseline age as the common time-independent covariates that influence the growth curve estimates in the analysis for all the cardiometabolic traits, and include statin use when fitting the model for lipids. The manifest variables (observed in the study) are in the rectangle, and the latent variables (not observed) are in the circle, double head arrows are variance or covariance of variables, single head arrows are either factor loading (from latent variable to manifest variable) or regressions. “mval” is the abbreviation for methylation value and “rf” is the abbreviation of risk factor. mval.IPT3 to mval.IPT9 mean the observed level of DNA methylation at different time points for one specific CpG site, and rf.IPT3 to rf.IPT9 represent the observed level of one specific cardiometabolic trait at different time points. mval.i and mval.s represent the intercept and slope of the growth model of DNA methylation on one specific CpG site, rf.i and rf.s represent the intercept and slope of the growth model of the trait. Since we fit a linear growth model for both DNA methylation and cardiometabolic traits, the factor loading from latent intercept to manifest v [file 13148_2021_1113_MOESM20_ESM.png]
